# Humid and Thermal Oxidative Ageing of Radiation Cured Polymers—A Brief Overview

**DOI:** 10.3389/fchem.2021.797335

**Published:** 2022-01-10

**Authors:** Xavier Colin

**Affiliations:** PIMM, Arts et Métiers Institute of Technology, CNRS, CNAM, HESAM University, Paris, France

**Keywords:** radiation cured polymer, humid ageing, water absorption, thermal oxidative ageing, changes in macromolecular skeleton, structure-property relationships, changes in mechanical properties

## Abstract

This article deals with the long-term behaviour of radiation cured polymers. Among the wide variety of possible ageing modes, the attention is focused on two key processes for users of radio-cured polymers: humid ageing of polymer glasses and thermal oxidative ageing of rubbers. These two processes are illustrated by numerous results coming from literature or our own research works. In both cases, the consequences of the structural modifications on the use properties (in particular, on mechanical properties) are described. It is found that the ageings of radiochemically and thermally cured polymers are not so different. It is thus concluded that a great part of the very abundant literature published on the ageing of thermally cured polymers remains exploitable for radio-cured polymers.

## Introduction

Radiation cured polymers became very attractive from the early 1970s because these polymers offer several advantages over thermally cured polymers such as: much faster curing, low energy requirements, room temperature treatment, non-polluting and solvent-free formulations, etc. They find many applications as protective coatings, inks, paints, varnishes and matrices for composite materials in various industrial sectors such as: electronics, dental restorative surgery, restoration of works of art, aeronautics, etc.

The main research works available in the literature on these polymers were mainly focused on their crosslinking reaction and kinetics (e.g., Crivello et al., 1999; Bowman and Kloxin, 2008; Coqueret, 2008; Ferry et al., 2016; Ghobashy, 2018). It was observed very early that the ionizing radiations lead to highly crosslinked and heterogeneous networks (Dušek, 1971; Dušek et al., 1980; Di Lorenzo and Seiffert, 2015). Thanks to the recent development of experimental techniques for probing materials at the nanoscale, it is now possible to more accurately study and analyze the structural characteristics of these networks. A general consensus for the description of the resulting microstructure consists in juxtaposing highly crosslinked nanosized clusters that are interconnected by a continuous domain made of a more weakly crosslinked matrix (Krzeminski et al., 2010a; Krzeminski et al., 2010b; Kowandy et al., 2018).

In contrast, the literature on the ageing of radio-cured polymers is relatively poor and is limited to a few research works (e.g., Dorbe, 2000; Lopitaux, 2001). Fortunately, thanks to the huge amount of research works devoted to thermally cured polymers, the wide variety of possible ageing modes are now well known. In particular, it is usual to distinguish between physical and chemical ageing. Physical ageing includes structural relaxation by solvent absorption (including solvent stress cracking) and additive loss by migration. Chemical ageing includes thermal, photochemical, radiochemical, biochemical and chemical processes in reactive media. Of course, many ageing modes can occur simultaneously and involve or not coupling effects. An exhaustive presentation of all these processes would be out of the scope of this article.

In this article, our attention will be focused on two key processes for users of radio-cured polymers: humid ageing of polymer glasses and thermal oxidative ageing of rubbers. This choice can be justified as follows:

- In the case of glasses, the temperatures of use are, by definition, lower than the glass transition temperature (*T*_*g*_). Reactive species possibly formed in the polymer remain trapped for long times in the glassy matrix owing to their low molecular mobility. Chemical processes responsible for chemical ageing, as well as structural relaxation, are extremely slow when typically: *T ≤ T*_*g*_ *—70 K*, which is the case in most common applications where materials are used around ambient temperature. However, water (in vapour or liquid state) interacts with polar matrices, even at ambient temperature. The “primary” effects of water absorption, i.e., plasticization and swelling, can be reversible at low water activities. At high activities, however, for matrices of moderate to high polarity, these processes can induce irreversible damage under external mechanical loading or simply under internal stresses induced by differential swelling. These processes have risen up an abundant literature since the 1970s owing to their crucial importance in aerospace applications. Chemical water-polymer interactions (i.e., hydrolysis) can play an important role in the humid ageing of thermosets, such as unsaturated polyesters, where they induce a well-known phenomenon by users of boats or swimming pools: blistering. This problem will be briefly evoked, although, to our knowledge, it seems to be negligible in most of radio-cured glasses at ambient temperature.
- Radiochemically cured rubbers (polyethylene will be included in this family despite its thermoplastic behaviour) have a hydrocarbon structure of which the low polarity guarantees the absence of humid ageing, except in very specific cases such as water trees under high electric stresses. However, hydrocarbon polymer structures are sensitive to radical oxidation. Oxidation mechanisms and kinetics have risen up a vast literature in the past half century, but the lack of consensus on certain key points makes its exploitation difficult, especially for beginners. An interesting feature of radical oxidation reactions is that these latter can be inhibited by a small concentration (typically lower than 1 wt%) of pertinently chosen additives. A less interesting feature of radiation curing is that it can consume a significant quantity of antioxidants, decreasing thus the residual polymer stability and lifetime. The role of antioxidants and the effect of radiation curing on their concentration will also be examined.

All these points will be illustrated by numerous results coming from literature or our own research works.

## Humid Ageing of Radio-Cured Thermosets

### General Aspects

Water sorption data, recorded in the 20–100°C temperature and 0–100 % relative hygrometry (RH) intervals are available for some radio-cured glasses of the diacrylate type, containing higher functionality polyacrylates as crosslinking agents. The following characteristics were reported: Water equilibrium concentration in saturated atmosphere ranges generally between 2 and 5 wt%. It increases generally with temperature and with relative hygrometry. When data are available on the effect of irradiation dose (or conversion), e.g., in the case of an epoxy diacrylate (Dorbe, 2000), they reveal that hydrophilicity increases with dose (and presumably with conversion). For thin samples, sorption curves clearly display a horizontal asymptote, e.g., an equilibrium plateau, and seem to obey Fick’s law in their transient regime. At long term, however, hydrolysis takes place and can induce irreversible changes.

### Hydrophilicity Versus Structure. A Brief Overview

In first approaches, hydrophilicity (which can be represented by the water mass gain at equilibrium $W_{e}$ in saturated atmosphere or in immersion in pure water) was found to depend mainly on the nature and concentration of elementary groups present in the polymer, through a molar additive function (Barrie, 1968). Schematically, three main types of chemical groups can be distinguished (van Krevelen and Te Nijenhuis, 2009):

1) Non hydrophilic groups such as:

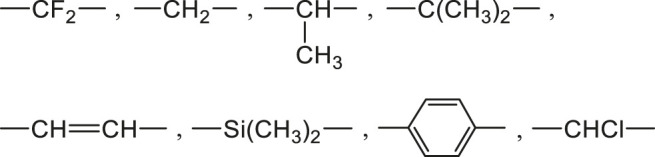


Polymers containing only these groups absorb generally less than 0.1 wt% of water, for instance: polyethylene (PE), polypropylene (PP), poly (tetrafluoro ethylene) (PTFE), poly (dimethyl siloxane) (PDMS), polybutadiene (PB), polystyrene (PS), poly (vinyl chloride) (PVC) and hydrocarbon rubbers.

2) Moderately hydrophilic groups such as:

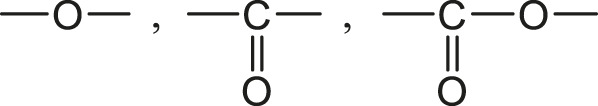


Polymers containing only these groups combined with groups of the family 1) absorb generally less than 3 wt% of water, for instance: poly (oxy phenylene) (PPO), poly (ether ether ketone) (PEEK), poly (methyl methacrylate) (PMMA), polycarbonate (PC), unsaturated polyesters (UP), poly (ethylene terephthalate) (PET) and poly (butadiene terephthalate) (PBT).

3) Highly hydrophilic groups such as:

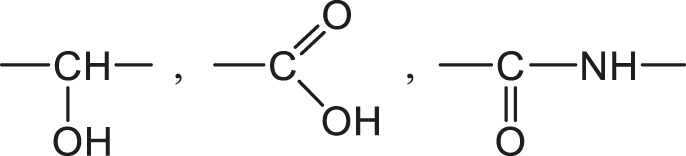


Polymers containing these groups in high concentration can be water soluble, for instance: poly (vinyl alcohol) (PVAl), poly (acrylic acid) (PAAc) and polyacrylamide (PAAm).

Starting from this observation, it was assumed the existence of a molar additive function that can be expressed as follows (Colin and Verdu, 2014):

$$H=\frac{W_{e}\times M}{1800}=\sum H_{i}$$ (1)

where *M* is the molar mass of the constitutive repeat unit (CRU) taken for the calculation and $H_{i}$ is the molar contribution of the *i*th group constituting the CRU, which is expressed in moles of water per mole of group.

It was attempted to apply this approach to a whole family of vinylester resins based on epoxy diacrylates, i.e., with a chemical structure very close to current radio-cured thermosets (Bellenger et al., 1994). Two hydrophilic structural units were distinguished:


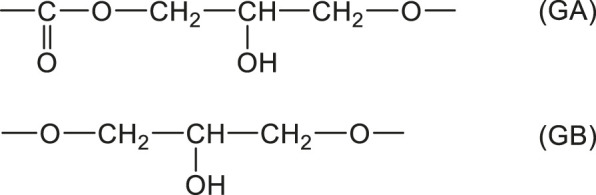


Correct predictions can be made taking H(GA)=0.356 and H(GB)=0.121 . These values could be used for rough estimations in the case of radio-cured thermosets, but there are many reasons to consider this result with caution:

- The fact that the molar contributions are not integers: Why 35.6 % of GA structures interact with one water molecule whereas 64.4 % do not interact?
- The fact that the molar contributions of similar structures are not constant within the same polymer family, for instance in amine crosslinked epoxies (Bellenger et al., 1989). In fact, it was found that $H_{i}$ is an increasing function of the group concentration (Tcharkhtchi et al., 2000).

A more sophisticated approach of hydrophilicity prediction will be proposed latter but here, one can already propose a rule of structural design to minimise hydrophilicity: Avoid groups of family (3) and increase the proportion of groups of family (1).

**FIGURE 1:**
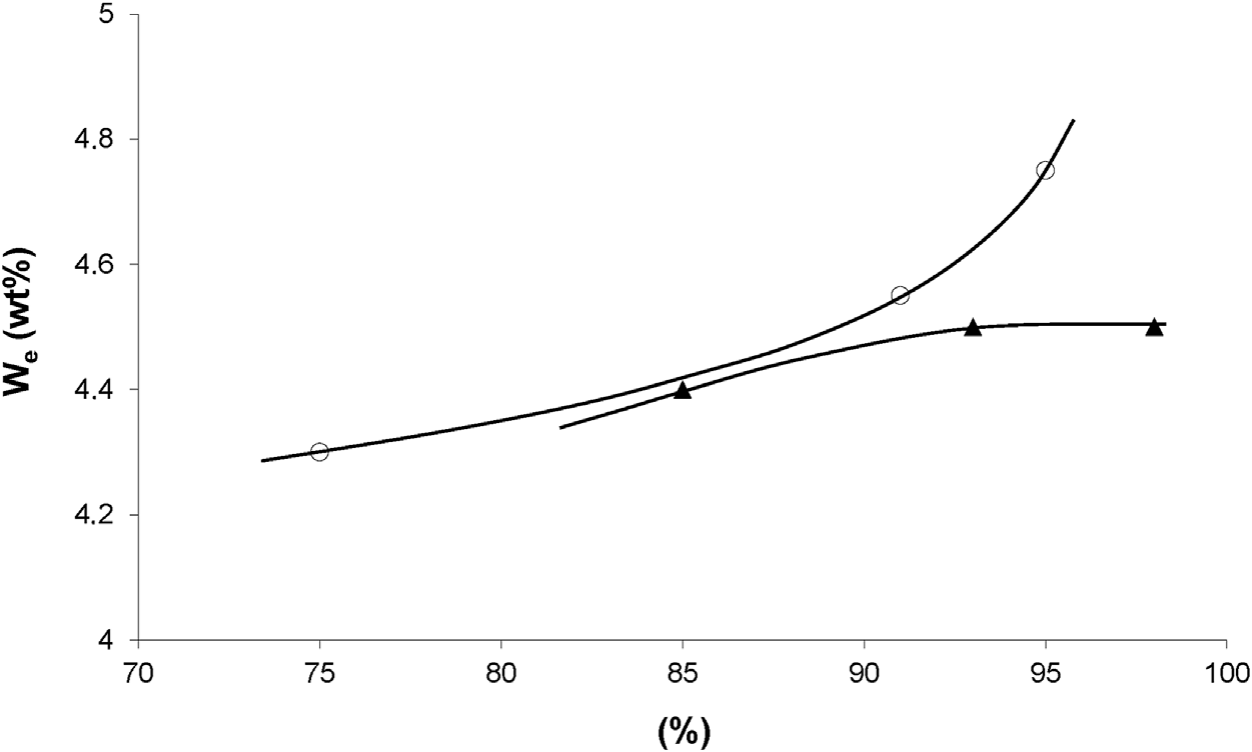
Equilibrium water mass uptake at 70°C under 100% RH versus conversion of acrylate groups during X rays (○) and electrons (▲) irradiation. Results coming from the PhD dissertation of Dorbe (2000).

Concerning irradiation effects on water sorption, we dispose of results for an epoxy acrylate system (Figure 1). For both modes of irradiation, water equilibrium concentration appears as an increasing function of conversion, possibly because saturated esters are more hydrophilic than unsaturated ones. But, the fact that both curves of Figure 1 diverge at high conversions indicates that other hydrophilic sites than esters could be involved, for instance groups resulting from radiochemical ageing at high doses (200 kGy).

For urethane based acrylic adhesives (AdUrMAc), hydrophilicity also appears to be a slightly increasing function of conversion (Lopitaux, 2001). At 70°C under 85% RH:

$$\frac{\Delta W_{e}}{\Delta \mathrm{conversion}}=5\times 10^{-2}$$ (2)

*W*_*e*_ and *conversion* being expressed in percent.

### Influence of Temperature on the Equilibrium Water Absorption

**FIGURE 2:**
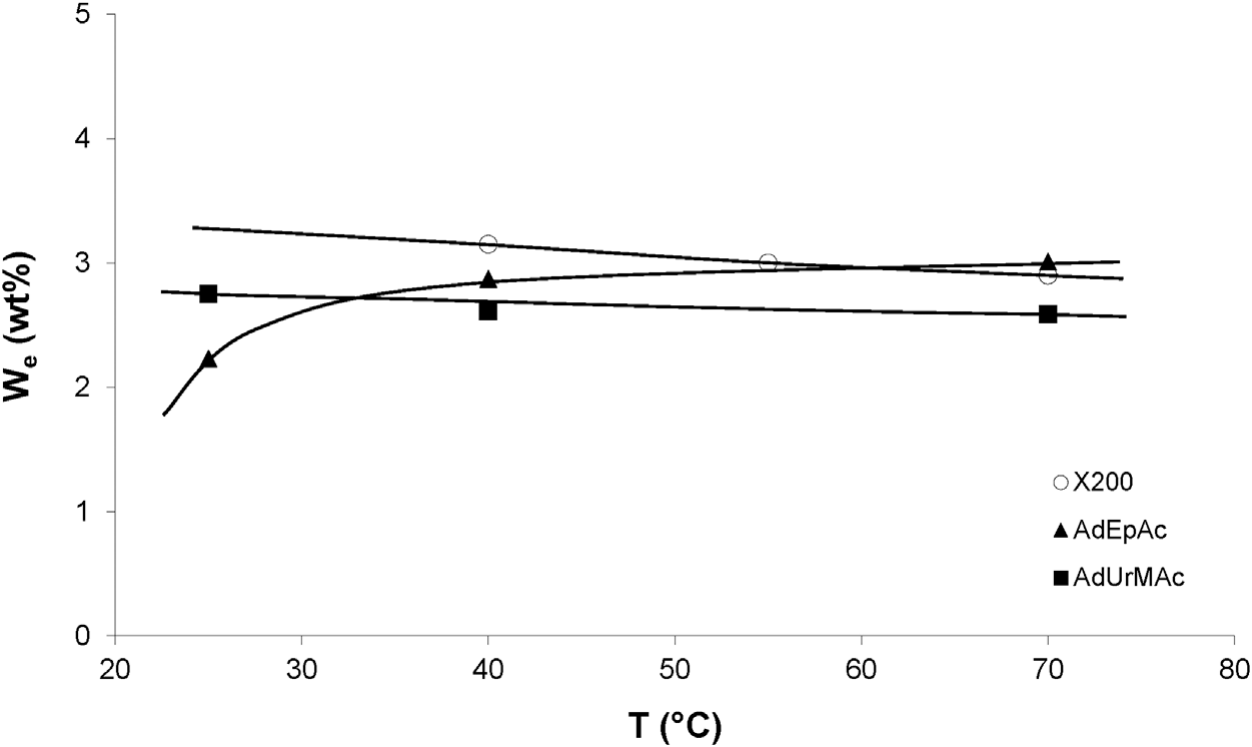
Influence of temperature on the equilibrium water mass uptake under 75% RH for X200 (Dorbe, 2000) and 80% RH for other samples (Lopitaux, 2001).

The temperature dependence of the equilibrium water concentration for various radio-cured thermosets is shown in Figure 2. These results can be summarized as follows. The water equilibrium concentration does not sharply depend on temperature, but it can be a decreasing or an increasing function of temperature, depending on the sample chemical structure. This result can be generalized as follows. $W_{e}$ appears as an increasing function of temperature, systematically in polymers containing groups of family (1) and (2), i.e., for low to moderate polarity, and a decreasing function of temperature in some polymers containing groups of family (3) in high concentration, i.e., for high polarity (Merdas et al., 2002).

These results can be explained by a simple thermodynamic analysis, considering, in a first approach, that the variation of the water equilibrium concentration *C* with the water vapour pressure *p* (in the case of humid atmosphere) or with the water vapour pressure in equilibrium with the bath (in the case of water immersion) can be represented by the Henry’s law:

C=p×S (3)

where

$$S=S_{0}\;\mathrm{Exp}\left(-\frac{H_{S}}{\mathrm{RT}}\right)$$ (4)

and

$$p=p_{0}\;\mathrm{Exp}\left(-\frac{H_{W}}{\mathrm{RT}}\right)$$ (5)

*H*_*S*_ and *H*_*W*_ being the heat of dissolution of water into the polymer and the heat of vaporization of water into the atmosphere (H_S_ ≈ 43 kJ mol^−1^), respectively.

It comes:

$$C=C_{0}\;\mathrm{Exp}\left(-\frac{H_{C}}{\mathrm{RT}}\right)$$ (6)

where

$$C_{0}=S_{0}\times \;p_{0}\;and\;H_{C}=H_{W}\times \;H_{S}$$ (7)

Since water-polymer interactions are an exothermic process, *H*_*S*_ is negative. Three cases can thus be distinguished:

- For low interactions: $\left|H_{S}\right|<H_{W}$ thus *H*_*C*_ *> 0* and *C* increases with temperature;
- For medium interactions: $\;\left|H_{S}\right|=H_{W}$ thus *H*_*C*_ *= 0* and *C* is independent of temperature;
- For strong interactions: $\;\left|H_{S}\right|>H_{W}$ thus *H*_*C*_ *< 0* and *C* decreases with temperature.

The three cases can be observed in Figure 2. It remains to explain two intriguing observations:

- Water-water interactions are extremely strong. How to explain the existence of stronger polymer-water interactions?
- In structural series differing only by the concentration of a given type of polar group, it was found that $\left|H_{S}\right|$ is an increasing function of the group concentration (Tcharkhtchi et al., 2000; Gaudichet-Maurin et al., 2008). In the simplest interpretation of the temperature dependence of S, only the pre-exponential factor is expected to depend on the group concentration. Indeed, the activation energy should only depend on the nature of the chemical group. How to explain such a dependence?

### Towards Refined Structure-Hydrophilicity Relationships

In order to explain the concentration dependence of the molar contribution $H_{i}$ and the heat of dissolution *H*_*S*_, it was suggested that a hydrophilic site would be a pair of polar groups rather than a single group (Gaudichet-Maurin et al., 2008). In other words, water molecules would be doubly bonded and, in order to play the role of hydrophilic site, a pair of polar groups should be separated by a distance compatible with this double bonding. Thus, hydrophilicity would be controlled by two characteristics: the distribution of distances between polar groups (indeed, the higher the concentration, the higher the fraction of close pairs) and the form *U(r)* of the hydrogen bond potential.

Schematically, hydrogen bond distances can typically vary (in the case of O-H ↔ O interactions) between 1.4–1.5 Å and 2.2–2.4 Å. Only pairs of polar groups that can establish bond distances within this interval with a water molecule will act as hydrophilic sites. This theory explains well the dependence of *H*_*S*_ with the polar group concentration and the fact that *H*_*S*_ values are so high. Indeed, *H*_*S*_ corresponds to two hydrogen bonds.

### Influence of Water Activity on Equilibrium Concentration

The above theories start from the assumption that the water equilibrium concentration (or mass gain $W_{e}$ ) obeys Henry’s law, i.e. is proportional to water activity *a*:

$$a=\frac{\mathrm{RH}}{100}$$ (8)

In fact, in the case of radio-cured thermosets, a deviation from linearity was observed. It is generally expressed in the mathematical form of a power law (Dorbe, 2000; Lopitaux, 2001):

$$W_{e}=B\times a^{n}$$ (9)

**FIGURE 3:**
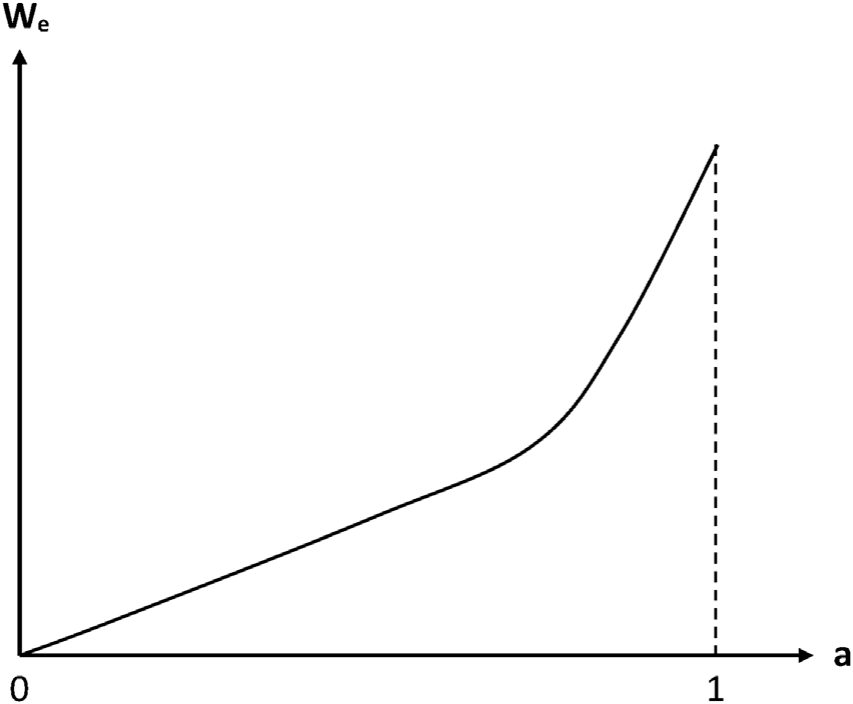
Schematization of the general trend of a sorption isotherm displaying no Langmuir component (Colin and Verdu, 2014).

Some values of parameters *B* and *n* are reported in Table 1. *n* values are ranged typically between 1 and 2. In other words, sorption isotherms display a slight positive curvature. However, detailed analyses of continuously recorded sorption isotherms indicate that the power law is probably not the most relevant relationship. Indeed, sorption isotherms display a linear shape in the domain of low activities and a more or less marked positive curvature in the domain of high activities (Figure 3).

**TABLE 1:** Parameters of the power law relating the equilibrium water concentration (expressed in wt%) to water activity *a = RH/100*.

Polymer	B	n	Source
AdEpAc	4.3	1.9	Lopitaux (2001)
AdUrMAc	3.3	1.1	Lopitaux (2001)
E or X	4.5	1.8	Dorbe (2000)

In such cases, it would be better to represent the water concentration by the sum of linear and power terms (Colin and Verdu, 2014):

$$W_{e}=H\times a+B\times a^{n}$$ (10)

Let us for instance consider the epoxy acrylate adhesive (AdEpAc) studied by Lopitaux (2001). Its characteristics at 70°C are given in Table 2.

**TABLE 2:** Calculation of the equilibrium water concentration at 70°C for AdEpAc using Eq. 10 with *H = 2*, *B = 3* and *n = 4*. Comparison to experimental data.

a	Experimental W_e_ (wt%)	Theoretical W_e_ (wt%)
0.95	3.79	4.34
0.8	3.01	2.83
0.5	1.17	1.19

No doubt, the fitting of experimental data is perfectible but, anyhow, it is not necessarily better than the power law. The advantage of Eq. 10 is that it can be physically interpreted. Indeed, if the deviation from linearity is due to the formation of water clusters, then the Zimm and Lundberg’s theory can be used to determine the cluster characteristics (Zimm and Lundberg, 1956; Lundberg, 1969; Starkweather, 1975). Let us consider the water saturation (i.e., when *a* = 1) for which this theory leads to fairly simple relationships, in particular:

$$W_{e}=W_{S}=H+B$$ (11)

According to the values obtained for parameters *B* and *n* by fitting experimental data (see Table 2), the cluster size at saturation (i.e., maximum number of water molecules in the cluster) would be:

$$\mathrm{CS}=\left(1-W_{S}\right)\left[1+\frac{\left(n-1\right)}{W_{S}}B\right]=2.66$$ (12)

In other words, the average number of water molecules per cluster at saturation would be of the order of 2 or 3. This cluster size is too small to be assimilated to a phase separation. In contrast, this latter can occur in porous samples where each pore becomes a water “micro-pocket”, detectable for instance by differential scanning calorimetry (DSC) since ice melting or water solidification gives respectively an endotherm or exotherm at 0°C, or at lower temperature in the case of smaller pores where confinement effects are significant. Indeed, expansion due to volume change at solidification during cooling or due to vaporization during heating at high rate can induce a stress state susceptible to initiate an irreversible damage.

The effect of structure on clustering is not well understood to our knowledge. In the same way, little is known on the effect of clustering on the physical properties of polymers. What is sure is that large clusters, i.e., free water forming a separated phase, do not participate to polymer plasticization (Colin and Verdu, 2014).

### Effect of Sorbed Water on the Glass Transition Temperature

The effect of water dissolved in a polymer on the glass transition temperature *T*_*g*_ can be theoretically predicted from two basic physical theories: entropy or free volume. Only this latter will be briefly presented here.

A given compound *i* is characterised by its glass transition temperature *T*_*gi*_, and its expansion coefficients *α*_*gi*_ and *α*_*li*_ in glassy and liquid/rubbery states, respectively. The expansion coefficient of free volume *α*_*i*_ is defined by:

$$\alpha_{i}=\alpha_{\mathrm{li}}-\alpha_{\mathrm{gi}}$$ (13)

The simplest free volume theory states that:

- For miscible mixture, the free volume components are additive,
- And the glass transition is an iso-free volume state.

For a given polymer (*T*_*gp*_, *α*_*p*_)-solvent (*T*_*gS*_, *α*_*S*_) mixture, the above assumptions lead to (Kelley and Boyer, 1961):

$$T_{g}=\frac{v_{P}\times \alpha_{P}\times T_{\mathrm{gP}}+v_{S}\times \alpha_{S}\times T_{\mathrm{gS}}}{v_{P}\times \alpha_{P}+v_{S}\times \alpha_{S}}$$ (14)

where $v_{P}$ and $v_{S}$ are the respective volume fractions of polymer and solvent ( $v_{S}=1-v_{P}$ ). This relationship simplifies using the Simha-Boyer’s rule (Simha and Boyer, 1962):

$$\alpha \times T_{g}=\mathrm{constant}\approx 0.113$$ (15)

It Comes:

$$\frac{1}{T_{g}}=\frac{1}{T_{\mathrm{gP}}}+A\times v_{S}$$ (16)

where

$$A=\frac{1}{T_{\mathrm{gS}}}-\frac{1}{T_{\mathrm{gP}}}$$ (17)

It can be seen that the plasticizing effect of a solvent (i.e., decrease in *T*_*g*_) is an increasing function of its volume fraction, and a decreasing function of *T*_*gS*_. For a given solvent, at a given volume fraction, the plasticizing effect is an increasing function of *T*_*gP*_.

The glass transition temperature *T*_*gS*_ of water has been controversial for a long time, but it is now well recognised that it is of the order of 120–150 K.

For a typical radio-cured polymer glass (*T*_*gp*_ ≈ 400 K and $v_{S}\approx 0.003$ ), it comes:

$$A\approx 5\times 10^{-3}K^{-1}$$ (18)

In this specific case, plasticization will cause the *T*_*g*_ of the polymer to drop by 8 K per weight percent of water absorbed. Unfortunately, in radio-cured thermosets, this plasticizing effect of water can be masked by post-curing effects.

### Effect of Sorbed Water on Volumetric Properties

Sorbed water induces a swelling. In contrast, water present in pores has no effect on the whole volume. Two extreme cases can be considered:

1) In the absence of pores, the totality of water is dissolved into the polymer, water and polymer volumes are additive. If the weight fraction of water in the mixture is *m*, it can be written (Belan et al., 1997):

$$\frac{1}{\rho }=\frac{1}{\rho_{P}}+\left(\frac{1}{\rho_{S}}-\frac{1}{\rho_{P}}\right)m$$ (19)

i.e.,

$$\frac{\rho }{\rho_{P}}=\frac{1}{1+\left(-1\right)m}$$ (20)

or

$$\frac{v}{v_{P}}=1+\left(\frac{\rho_{P}}{\rho_{S}}-1\right)m$$ (21)

where v is the specific volume and ρ is the density. Indexes P and S denote the polymer and solvent, respectively. One can see that the swelling effect is an increasing function of the density ratio $\rho_{P}/\rho_{S}$ .

2) The totality of water is present in pores. No water is dissolved in the polymer. In this case, if *m* is the mass uptake linked to water absorption:

$$\rho =\rho_{P}\left(1+m\right)=\rho_{P}\left(1+u\frac{\rho_{S}}{\rho_{P}}\right)$$ (22)

i.e.

vvP=11+u ρSρP (23)

where *u* is the pore volume fraction in the polymer.

Thus, here, the density increases (or the specific volume decreases) when increasing the volume fraction of pores.

Let us recall that the presence of free water in pores can be detected by differential scanning calorimetry (DSC), nuclear magnetic resonance spectroscopy (NMR) or dielectric spectroscopy, for instance.

Equations 21, 23 can be considered as the upper and lower boundaries of the volume variation domain, respectively. In addition, even in the absence of pores, the existence of strong water-polymer interactions can induce a volume contraction so that the swelling is lower than predicted from Eq. 21 (Belan et al., 1995).

### Water Diffusivity

Diffusion of water in radio-cured thermosets of moderate hydrophilicity seems to obey Fick’s law within experimental scattering. Diffusion coefficient values are almost independent of water activity. In contrast, they increase with temperature. Some typical values are reported in Table 3.

**TABLE 3:** Diffusivity of water in various radio-cured acrylic matrices. Results coming from the PhD dissertations of Dorbe (2000) and Lopitaux (2001).

Temperature (°C)	RH (%)	D × 10^12^ (m^2^.s^−1^)					
40	100	0.69	0.44	0.72	0.47	—	—
40	80	—	—	—	—	0.24	0.81
70	100	6.91	2.93	4.76	2.71	—	—
70	80	—	—	—	—	2.17	4.39
Activation energy (kJ.mol^−1^)		—	—	—	50	46	44
Material		X50	X200	E50	E200	AdEpAc	AdUrMAc
Source		Dorbe (2000)				Lopitaux (2001)	

These data call for the following comments:

1) For the systems under investigation, the diffusion coefficient values are of the same order of magnitude, e.g., about 10^−12^ m^2 ^s^−1^ at 70°C.
2) The apparent activation energies are also of the same order of magnitude, i.e., about 45–50 kJ mol^−1^.
3) In the epoxy-acrylate system studied by Dorbe (2000), *D* appears to be a decreasing function of irradiation dose, i.e. of conversion ratio. At 70°C, especially in wet state, these matrices are within their glass transition temperature domain, with cooperative mobilities sharply depending on the gap: $T=T_{g}-70{}^{\circ}C$ *.* Since the higher the conversion ratio, the higher the *T*_*g*_ and thus, the higher this gap, it is not surprising to observe a decrease in *D* with the conversion ratio.

Concerning structure-diffusivity relationships, it is often claimed that *D* depends essentially on the free volume fraction. However, in many structural series, it is systematically observed that *D* is a decreasing function of the equilibrium water concentration (Thominette et al., 2006), which seems to indicate that diffusion is slowed down by the strong water-polymer interactions (i.e., hydrogen bonds). Water diffusion mechanisms and kinetics (in polymers) remain, to our opinion, a widely open research domain. In unidirectional composites, water diffusivity becomes anisotropic. In a first approach, by analogy with thermal diffusion, it can be written (Colin and Verdu, 2012):

$$D_{∥}=\left(1+v_{f}\right)D$$ (24)

and

$$D_{⊥}=\left(1-2\sqrt{\frac{v_{f}}{\pi }}\right)D$$ (25)

where $v_{f}$ is the volume fraction of fibres (assumed to be impermeable to water); $D_{∥}$ and $D_{⊥}$ are the diffusion coefficients in parallel and perpendicular directions to the fibres, respectively.

Here, swelling effects can play a key role in damage at long-term, that explains the abundance of literature works on water diffusion kinetics in composites (Colin and Verdu, 2012; Colin and Verdu, 2014).

### Hydrolytic Ageing

#### Shape of Gravimetric Curves

**FIGURE 4:**
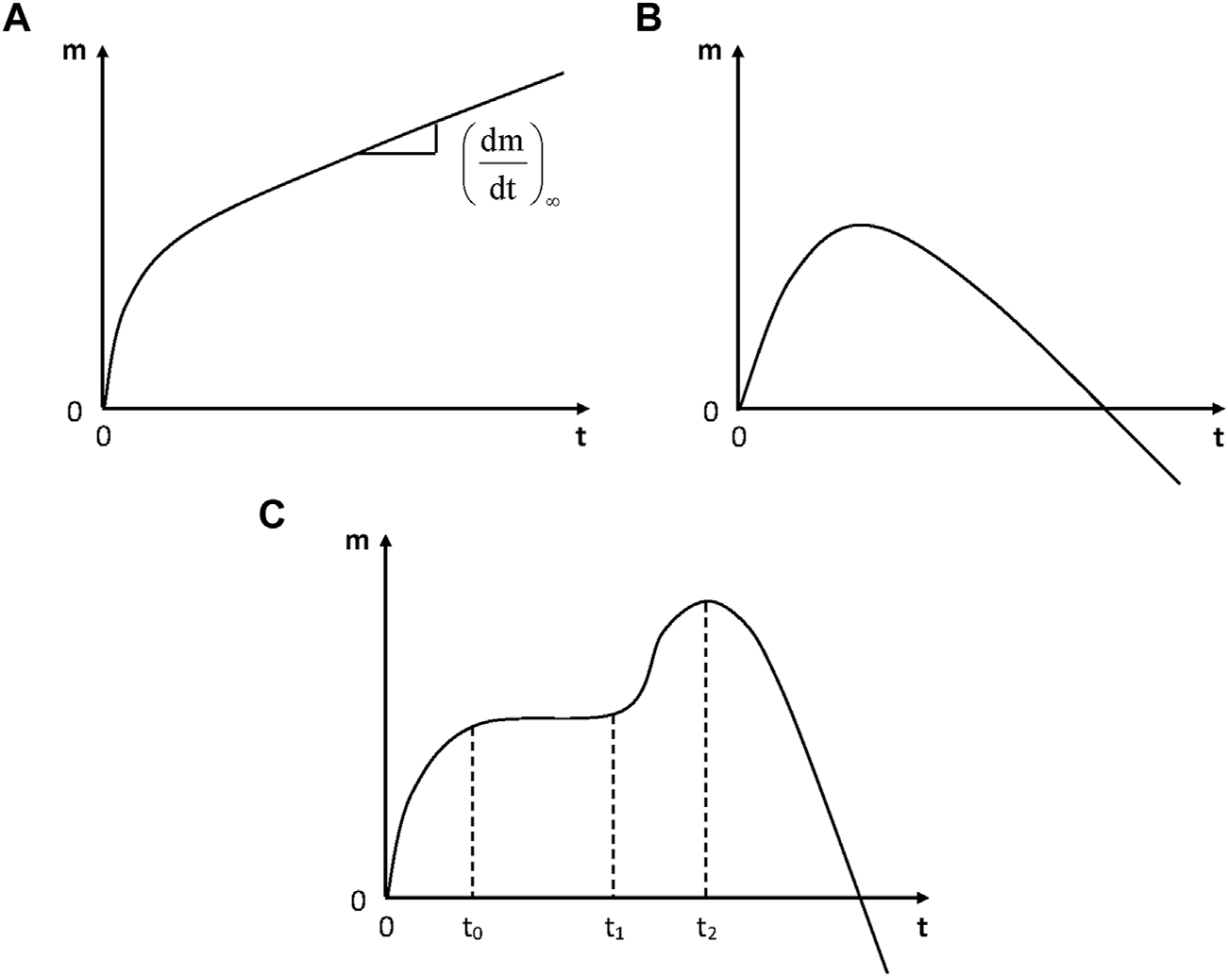
Schematization of the general trends of gravimetric curves for the hydrolytic aging of polymers. **(A)** Simple case: incorporation of water molecules into the polymer structure; **(B)** Predominance of weight loss at longer term; **(C)** Hydrolysis coupled with osmotic cracking.

In the absence of irreversible damage, physical humid ageing is characterized by the existence of a sorption equilibrium and by the fact that the molar mass (in linear polymers) or the crosslink density (in networks) remains unchanged. In some cases, however, continuous weight changes are observed whereas no visible damage appears. Weight changes can be positive or non-monotonic (Figure 4).

In the simplest case (Figure 4A), the weight increase would only be due to the incorporation of water in the polymer structure, for instance in the case of a polyamide (El Mazry et al., 2012):


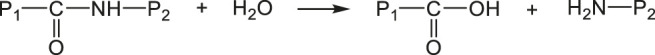


If the change in hydrophilicity due to hydrolysis is negligible or if the sample is weighed after drying, a simple relationship is expected between the (steady) rate of mass increase ${\left(dm/dt\right)}_{\infty }$ and the hydrolysis rate (dS/dt) :

$$\frac{\mathrm{dS}}{\mathrm{dt}}=\frac{1}{1800}{\left(\frac{\mathrm{dm}}{\mathrm{dt}}\right)}_{\infty }$$ (26)

where *S* is the number of chain scissions (expressed in mol.g^−1^) and *m* the mass uptake in weight percent.

However, this is an ideal case. Two cases of deviation from ideality are often observed:

1) Hydrolysis induces a strong increase in hydrophilicity. As an example, ester hydrolysis leads to the replacement of a moderately hydrophilic ester group by two highly hydrophilic groups: one acid and one alcohol. Then, there is a great difference between wet and dry states for a given aged sample and thus, the above equation would overestimate the rate of chain scissions.
2) The yield of small (extractible and/or volatile) molecular fragments resulting from hydrolysis is not negligible. The corresponding weight loss can predominate over weight uptake due to hydrolysis, and the curves can display the shape of Figure 4B. Radio-cured epoxy acrylates belong to ester family. In such materials, hydrolysis can be monitored from the disappearance of ester groups, for instance using their near IR band at 6164 cm^−1^ (Dorbe, 2000), or their IR bands at 1730 cm^−1^ and 1410 cm^−1^, or their NMR bands at 166 ppm for residual acrylates and 175 ppm for the ester of reacted (saturated) acrylates. Typical values of the pseudo first order rate constant K determined from the rate of ester consumption are reported in Table 4.

**TABLE 4:** Pseudo first order rate constant for ester consumption at 70°C under various relative hygrometries. Results coming from the PhD dissertations of Dorbe (2000) and Lopitaux (2001).

Sample	Dose (kGy)	Conversion (%)	Temperature (°C)	Hygrometry (%)	K × 10^6^ (h^−1^)	Source
AdEpAc	35	≈48	70	65	40	Lopitaux (2001)
AdEpAc	35	≈48	70	100	96	Lopitaux (2001)
AdEpAc	100	≈60	70	65	34	Lopitaux (2001)
AdEpAc	100	≈60	70	100	83	Lopitaux (2001)
E50	50	75–85	70	55	36–39	Dorbe (2000)
E100	100	91–93	70	100	80–103	Dorbe (2000)
E50	50	75–85	55	55	5	Dorbe (2000)
E100	100	91–93	55	100	16–19	Dorbe (2000)

The results of both authors are in good agreement showing that epoxy-acrylates investigated were structurally close if not identical. K values indicate a noticeable reactivity of epoxy acrylates. For methacrylic vinylesters, for instance, values of 50 × 10^−6^ h^−1^ were found (Ganem et al., 1994; Bellenger et al., 1995), whereas for unsaturated polyesters, K ranges between 30 and 200 × 10^−6^ h^−1^ at 70°C under 100% RH (Belan et al., 1997). In other words, radio-cured epoxy acrylates are not fundamentally different from other aliphatic polyesters and more reactive than methacrylates.

The effect of radiation dose, i.e., conversion, is noticeable. The reactivity to hydrolysis is a decreasing function of conversion, that could be attributed to the fact that unsaturated esters are more reactive than saturated ones, whereas these latter are more hydrophilic.

Finally, it is interesting to note the strong influence of relative hygrometry. Radio-cured epoxy acrylates studied by Dorbe (2000), which were exposed up to 14 000 h at 70°C under 100% RH, display the shape of Figure 4A in wet state and the shape of Figure 4B in dry state, thus showing a considerable increase in hydrophilicity due to hydrolysis.

In some cases (Figure 4C), hydrolysis is coupled with osmotic damage. Typical gravimetric curves reveal the existence of a physical sorption equilibrium (at time *t*_*0*_), the onset of osmotic cracking (*t*_*1*_) and the crack coalescence (*t*_*2*_) followed by a fast mass decrease. The following explanation was proposed (Gautier et al., 1999): hydrolysis generates small molecular fragments (diols, diacids, etc.) which remain trapped in the matrix and accumulate until they reach their solubility limit. Then, they demix forming highly hydrophilic liquid micropockets that will act as crack nuclei. Then, cracks will propagate due to the build-up of osmotic pressure owing to the fact that the polymer acts as a semi-permeable membrane separating the cracks from the bath. When cracks coalesce, the quasi-totality of organic molecules pass in the bath and the mass decreases rapidly. If the sample thickness is too high, both sorption and osmotic cracking cannot be decoupled and the gravimetric curves have the shape of Figure 4B.

#### Hydrolysis Mechanisms and Kinetics

Hydrolysis is always an ionic mechanism, catalysed by ions H^+^ or OH^−^. The effect of acids or bases on hydrolysis rate is not easy to interpret because ionic species are generally insoluble in polymers. What penetrates in the polymer is the undissociated acid (or basis) (Ravens, 1960). Hydrolysis is more or less reversible (e.g., Jacques et al., 2002):


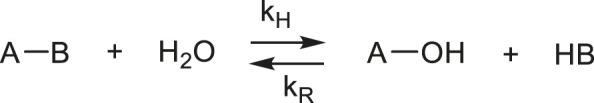


The equilibrium occurs when:

$$\frac{\left[\mathrm{AOH}\right]\times \left[\mathrm{BH}\right]}{\left[\mathrm{AB}\right]\times \left[H_{2}O\right]}=\frac{k_{H}}{k_{R}}$$ (27)

In the case of polyesters, the equilibrium occurs generally at a relatively high conversion, often after the mechanical properties have been completely lost. In such cases, the condensation reaction can generally be neglected (e.g., McMahon et al., 1959).

In the case of polyamide 11, for instance, the equilibrium occurs at a very low conversion and the reverse reaction cannot be neglected (Jacques et al., 2002; El Mazry et al., 2012). In the simplest case, the pseudo first order rate constant *K* is proportional to water concentration:

Ester + Water ↔ Products (k_H_) (No Reverse Reaction)

$$\frac{d\left[E\right]}{\mathrm{dt}}=-k_{H}\left[E\right]\left[W\right]$$ (28)

where [*E*] and [*W*] are the respective ester and water concentrations.

$$K=-\frac{1}{\left[E\right]}\frac{d\left[E\right]}{\mathrm{dt}}=k_{H}\left[W\right]$$ (29)

**FIGURE 5:**
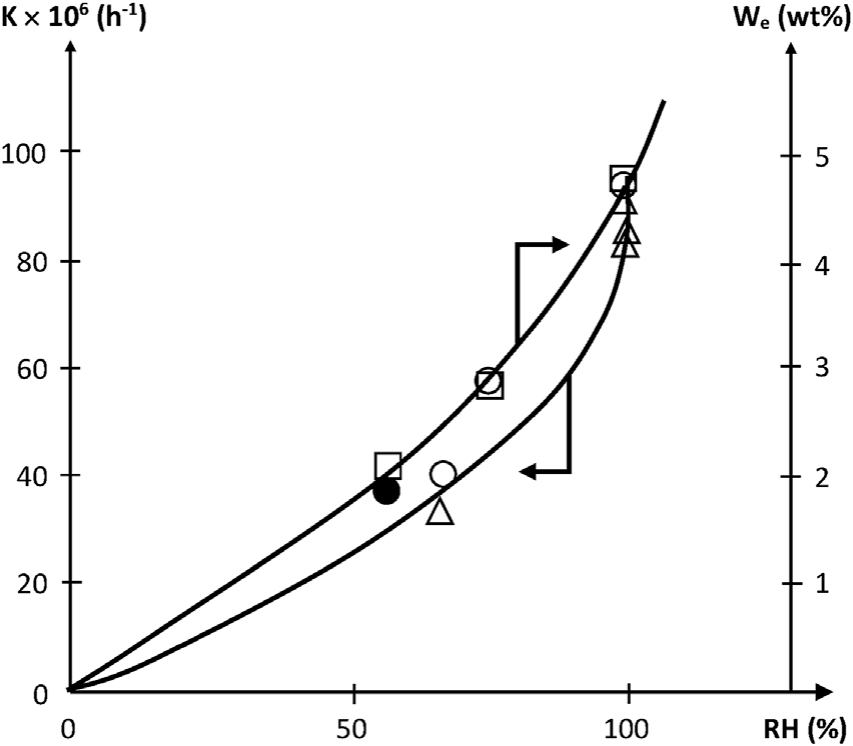
Variation of the pseudo first order rate constant of acrylate hydrolysis and of equilibrium water uptake with relative hygrometry at 70°C. □ Dorbe’s results on the water equilibrium concentration (Dorbe, 2000). ○ △ ● Dorbe and Lopitaux results on the hydrolysis rate constant (Dorbe, 2000; Lopitaux, 2001).

Since [*W*] tends to increase quasi parabolically with *RH*, *K* is expected to display the same trend, as effectively observed (Figure 5).

## Thermal Oxidative Ageing of Radio-Cured Rubbers

### Introduction

Hydrocarbon polymer substrates react with atmospheric oxygen through radical chain processes of which the main mechanistic features were established just after the second world war (Bolland and Gee, 1946a; Bolland and Gee, 1946b). The most general form of the mechanistic scheme could be the following:

Initiation:

1) *δ*Per → *α*P^•^ + *β*PO_2_^•^ (*k*_*1b*_) (with Per = peroxide or hydroperoxide)

Propagation:

2) P^•^ + O_2_ → PO_2_^•^ (*k*_*2*_)
3) PO_2_^•^ + Sub → Per + P^•^ (*k*_*3*_) (with Sub = substrate; H abstraction or addition to double bond)

Terminations:

4) P^•^ + P^•^ → Inactive products (*k*_*4*_)
5) P^•^ + PO_2_^•^ → Inactive products (*k*_*5*_)
6) PO_2_^•^ + PO_2_^•^ → Inactive products + O_2_ (*k*_*6*_)

The corresponding kinetic scheme must be composed of at least 5 differential equations corresponding to the 5 reactive species: P^•^, PO_2_^•^, Per, Sub and O_2_. For O_2_, the corresponding differential equation must contain a diffusion term because it is experimentally observed that oxidation is diffusion limited in most practical cases (except for thin films of about 100 µm thick). The set of differential equations can be used to predict the ageing characteristics provided that the 6 rate constants and the 5 initial concentrations of the reactive species are known.

This system (and a fortiori, more complex systems with, for instance, coexistence of many initiation modes or coexistence of H abstraction and PO_2_^•^ addition to double bonds) cannot be analytically solved, except if simplifying assumptions are made. The most common assumptions are the following ones:

U– Unicity of the reactive site. The complexity of the scheme increases exponentially with the number of reactive sites
S– Steady rate for radical concentration
E– Oxygen excess (in this case, reactions (5) and (6) can be neglected)
C– Constancy of the substrate concentration (i.e., low conversions)
I– Constancy of the initiation rate
H– Stability of peroxides
T– Relationship between termination rate constants, i.e.:

$$k_{5}^{2}=4k_{4}\times k_{6}$$ (30)

L– Long kinetic chains, i.e. propagation rate >> initiation rate.

The history of oxidation kinetics, in the past 60 years, can be described as the history of attempts to progressively suppress all these assumptions. All the above assumptions together lead to the simplest kinetic scheme (called Scheme A below) which can formally be reduced to only three reactions. Note that, in Scheme A, H abstraction can be replaced by PO_2_^•^ addition to double bonds. But, in this case, hydroperoxides POOH have to be replaced by peroxides POOP. The resulting oxidation kinetics remains globally the same.

#### Scheme A

Initiation:

Polymer (or initiator) + O_2_ → PO_2_^•^ (*r*_*i*_)

Propagation:

PO_2_^•^ + PH + O_2_ → POOH + PO_2_^•^ (*k*_*3*_)

Terminations:

PO_2_^•^ + PO_2_^•^ → Inactive products + O_2_ (*k*_*6*_)

It can be easily shown that, according to the above 8 assumptions:

$$r_{\mathrm{ox}}=-\frac{d\left[O_{2}\right]}{\mathrm{dt}}=k_{3}\left[\mathrm{PH}\right]{\left(\frac{r_{i}}{2k_{6}}\right)}^{1/2}$$ (31)

It appears that the oxidation rate depends mainly of two quantities:

1) The initiation rate *r*_*i*_ which can be considered as an extrinsic quantity. This rate is related to the radiation intensity in the case of irradiation, to the nature and concentration of initiator in the case of chemical initiation, etc.
2) The ratio $k_{3}/\sqrt{k_{6}}$ , which can be considered as an intrinsic characteristic of the substrate structure. This ratio will appear in all the other kinetic schemes. In literature, it was called the substrate “oxidizability”.

Use of radical initiators (peroxides, azobisisobutyronitrile, etc.) is pertinent provided that, in the exposure conditions, initiators are decomposed with a rate considerably higher than the peroxides/hydroperoxides resulting from the substrate oxidation. Separate determinations of *k*_*3*_ and *k*_*6*_ are extremely difficult. Fortunately, the determination of the “oxidizability ratio” $k_{3}/\sqrt{k_{6}}$ is relatively easy from the measurements of the steady rate of oxygen consumption $r_{\infty }$ :

$$\frac{k_{3}}{\sqrt{k_{6}}}=\frac{\sqrt{2}\times r_{\infty }}{\left[\mathrm{PH}\right]\times \sqrt{r_{i}}}$$ (32)

**FIGURE 6:**
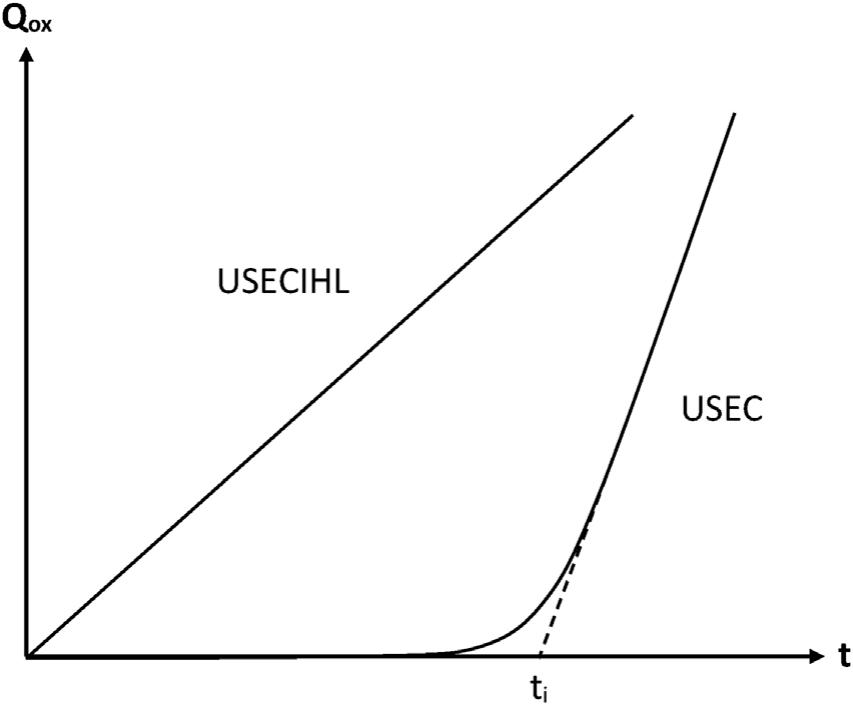
Schematization of the general trend of the oxidation kinetic curves (*Q*_*ox*_ = quantity of consumed oxygen) for the sets of assumptions indicated in this figure. Note that, in USEC, the assumptions relative to the constancy of initiation rate (I) and hydroperoxide stability (H) have been suppressed.

Chemically initiated oxidations are not representative of thermal oxidation because, in this latter case, the main source of radicals is the decomposition of hydroperoxides (or peroxides) (Colin et al., 2006; Colin et al., 2007a; Coquillat et al., 2007a; Colin et al., 2019). The difference of kinetic behaviour is obvious (Figure 6). In this case, the mechanistic scheme corresponds to Scheme B.

#### Scheme B

Initiation:

*δ*POOH + O_2_ → 2PO_2_^•^ (k_1_)

Propagation:

PO_2_^•^ + PH + O_2_ → POOH + PO_2_^•^ (k_3_)

Termination:

PO_2_^•^ + PO_2_^•^ → Inactive products + O_2_ (k_6_)

Only the bimolecular POOH decomposition (*δ* = 2) will be considered here because it is the most important at low temperatures (typically for T < 200°C). If the initial hydroperoxide concentration is low, the reaction begins slowly but, since hydroperoxides accumulate, the initiation rate increases and the reaction progressively auto-accelerates. As a result, the kinetic curves display an induction period of which the duration $t_{i}$ is a decreasing function of two quantities:

- The pseudo first order rate constant *K* defined by:

$$K=k_{3}\left[\mathrm{PH}\right]{\left(\frac{k_{1}}{k_{6}}\right)}^{1/2}$$ (33)

- And the initial hydroperoxide concentration: ${\left[POOH\right]}_{0}$ .

It can be shown that (Colin et al., 2006):

$$t_{i}=\frac{1+\mathrm{Ln}\left(b\right)}{K}$$ (34)

with

$$b=\frac{{\left[\mathrm{POOH}\right]}_{\infty }-{\left[\mathrm{POOH}\right]}_{0}}{{\left[\mathrm{POOH}\right]}_{0}}\;and\;{\left[\mathrm{POOH}\right]}_{\infty }=\frac{k_{3}\left[\mathrm{PH}\right]}{2{\left(k_{1}/k_{6}\right)}^{1/2}}$$ (35)

This kinetic scheme was established for the first time by Tobolsky et al. (1950) in the middle of the past century. Unfortunately, it was completely forgotten in the following decades, so that in the 1990s, it was claimed that modelling of such processes displaying an induction period is impossible.

In the 1960-70s, research efforts were essentially concentrated on the study of structure effects on the rate constants. The most striking event, from the kinetic point of view, is the detailed study of Scheme A by suppressing the assumption of long kinetic chain (L) by Mayo and co-workers (e.g. Decker et al., 1973; Decker and Mayo, 1973). Applications of Mayo’s results to radiochemically initiated oxidation at relatively high dose rates and relatively low temperatures are obvious (Khelidj et al., 2006a; Khelidj et al., 2006b)

Both above Schemes A and B have in common assumption E, i.e., oxygen excess, which is valid only in the case of thin samples and under high oxygen pressures, i.e., out of the working domain of most rubber applications. In these latter, it is well known that oxidation is diffusion limited, which means that assumption E should be suppressed. Solutions were proposed in the early 1980s (e.g., Cunliffe and Davis, 1982), but only in the case of a constant initiation rate (i.e. with assumptions I and H) and long kinetic chain (L), that finally leads to Scheme C.

#### Scheme C

Initiation:

Polymer (or initiator) → P^•^ (*r*_*i*_)

Propagation:

P^•^ + O_2_ → PO_2_^•^ (*k*_*2*_)
PO_2_^•^ + PH → POOH + P^•^ (*k*_*3*_)

Terminations:

P^•^ + P^•^ → Inactive products (*k*_*4*_)
P^•^ + PO_2_^•^ → Inactive products (*k*_*5*_)
PO_2_^•^ + PO_2_^•^ → Inactive products + O_2_ (*k*_*6*_)

Here, according to assumptions U, S, C, I, H, T and L, the local oxygen consumption rate is given by:

$$r_{\mathrm{ox}}=\frac{r_{S}\times r_{0}}{r_{S}+r_{0}}$$ (36)

with

$$r_{S}=k_{3}\left[\mathrm{PH}\right]{\left(\frac{r_{i}}{2k_{6}}\right)}^{1/2}\;\text{a}\text{n}\text{d}\;r_{0}=k_{2}\left[O_{2}\right]{\left(\frac{r_{i}}{2k_{4}}\right)}^{1/2}$$ (37)

This expression can be rewritten such as:

$$r_{\mathrm{ox}}=\frac{\alpha \left[O_{2}\right]}{1+\beta \left[O_{2}\right]}$$ (38)

where $\left[O_{2}\right]$ is the local oxygen concentration, while *α* and *β* are constants depending on elementary rate constants:

$$\alpha =k_{2}{\left(\frac{r_{i}}{2k_{4}}\right)}^{1/2}\;\text{a}\text{n}\text{d}\;\beta =\frac{k_{2}}{k_{3}\left[\mathrm{PH}\right]}{\left(\frac{k_{6}}{k_{4}}\right)}^{1/2}$$ (39)

In an elementary thickness layer, located at a distance z beneath the sample surface, the oxygen concentration balance is given by:

$$\mathrm{Changes}\;\mathrm{in}\;\;\left[O_{2}\right]=O_{2}\mathrm{supplied}\;\mathrm{by}\;\mathrm{diffusion}-O_{2}\mathrm{consumed}\;\mathrm{by}\;\mathrm{the}\;\mathrm{reaction}$$ (40)

i.e.

$$\frac{d\left[O_{2}\right]}{\mathrm{dt}}=D\frac{\partial^{2}\left[O_{2}\right]}{\partial z^{2}}-\frac{\alpha \left[O_{2}\right]}{1+\beta \left[O_{2}\right]}$$ (41)

where *D* is the coefficient of oxygen diffusion in the polymer.

This differential equation can analytically be solved in two limiting cases (Audouin et al., 1994):

- When $\beta \left[O_{2}\right]\ll 1$ :

$$r_{\mathrm{ox}}=\alpha \left[O_{2}\right]$$ (42)

- And when $\beta \left[O_{2}\right]\gg 1$ :

$$r_{\mathrm{ox}}=\alpha /\beta$$ (43)

The system is assumed to rapidly reach a steady state (i.e., $d\left[O_{2}\right]/dt=\;$ 0) so that the model is reduced to a simple second order differential equation capable of predicting the thickness profile of oxygen concentration, i.e., $\left[O_{2}\right]=f\left(z\right)$ . Since the relationship between oxidation rate and oxygen concentration is known, it is possible to determine the profile of oxidation rate and, by integration of this latter, to determine the profile of oxidation conversion. This latter can experimentally be checked by performing measurements on thin slices of microtome cut samples or using mapping methods (Gillen and Clough, 1989). In most cases, the thickness of the oxidized layer *TOL*, in which most oxidation products are concentrated, can be estimated using a simple scaling law (Audouin et al., 1994):

$$\mathrm{TOL}={\left(\frac{D\times \left[O_{2}\right]}{r_{\mathrm{ox}}}\right)}^{1/2}$$ (44)

It is noteworthy that at least one supplementary simplifying assumption was added:

D– Steady state in the coupled diffusion-reaction system.

With this set of assumptions, the model can be applied to radiochemically initiated oxidation at relatively high dose rates (> 0.1 Gy s^−1^). Gillen et al. (1995) used numerical methods to suppress assumption T (i.e., $k_{5}^{2}=4k_{4}\times k_{6})$ , which is, no doubt, an ad hoc assumption aimed at only facilitating calculations.

In the 1980s, Sommersall and Guillet (1985) published a very interesting attempt of numerical solving of a very complex scheme of photooxidation, suppressing all assumptions, except assumptions U and E (i.e., oxygen excess). Unfortunately, there was no continuation to this work suffering from the lack of experimental validation.

Research was also very active in the former USSR, especially in the domain of physical chemistry (Emanuel and Buchachenko, 1987). Some of the above models were also developed by Russian co-workers but their main discoveries seem to have been oriented rather on fundamental aspects than on the practical problem of lifetime prediction.

In the 1990s, the main novelty was the “infection model” proposed by Celina and George (George and Celina, 2000). This model is based on the idea that, in hydrocarbon polymers, oxidation propagates locally from initiation centres owing to the low molecular mobility of reactive species. Thus, oxidation invades progressively the sample volume as an epidemy propagating from infectious centres. In its initial form, however, this model has the mathematical form of a homogeneous kinetic model, i.e., the unique variable is the time, and it cannot take into account the induction phenomenon or the kinetic control by oxygen diffusion, so that it is too soon to determine if it is suitable for lifetime prediction. In the case of stabilised PP, it is well established that, after the end of induction time, oxidation effectively propagates heterogeneously by radical growth from a small number of “nucleation” centres (Fayolle et al., 2002a). However, embrittlement occurs before the end of the induction period, and during this latter, the molecular mass distributions are consistent with the assumption of a homogeneous degradation (Fayolle et al., 2002b).

In the two last decades, a revival of the classical kinetics was tentatively initiated in our laboratory, trying to suppress all assumptions except, for the time being, the assumption of unicity of the reactive site (U), and directly introducing the diffusion terms (oxygen, stabilisers) in the set of differential equations derived from the mechanistic scheme. Examples of application of this approach in the field of rubbers have been published, in particular for vulcanized polyisoprene (Colin et al., 2007a; Colin et al., 2007b; Colin et al., 2007c), polybutadiene (Coquillat et al., 2007a; Coquillat et al., 2007b), and ethylene-propylene-diene (EPDM) terpolymers (Colin et al., 2019). The coexistence of H abstraction and radical additions to double bonds (these latter being intra or intermolecular) and the existence of a variety of side reactions specific to the chosen vulcanization system (sulphur vulcanization) make these models extremely complex: one dozen of differential equations. Their validation needs a large number of distinct experiments: build-up of carbonyls and hydroxyls, consumption of double bonds, changes in Young’s modulus, weight changes, thickness distribution of oxidation products, etc. But, the number and diversity of validation tests guaranty the model reliability for lifetime prediction.

In some cases, stabilisation reactions and stabiliser migration have started to be introduced into the kinetic model (Coquillat et al., 2008; Colin et al., 2019). In a more remote future, diffusion of macromolecular species will also be taken into account in order to simulate eventual heterogeneous ageing phenomena. However, this requires solving non-trivial numerical problems of tri-dimensional diffusion.

### Network Structure and Rubbery Properties

Compared to glassy polymers, rubbers are first characterized by systematic differences in oxygen transport properties. Oxygen solubility and diffusivity are much higher in rubbers than in glassy polymers (van Krevelen and Te Nijenhuis, 2009).

**FIGURE 7:**
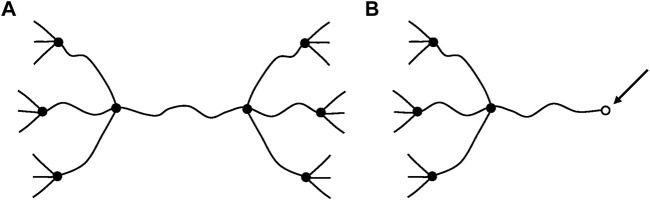
Schematization of a network with tetrafunctional crosslink nodes. **(A)** Ideal network. **(B)** Non-ideal network: it contains dangling chains with a free extremity (arrow).

Another very important difference is that, in glassy polymers, chain scissions or crosslinking reactions, at reasonably low conversions, have no significant effect on elastic properties. In contrast, in rubbers, chain scissions or crosslinking reactions have a direct consequence on tensile (*E*) or shear (*G*) elastic modulus. We dispose of a theoretical tool: the theory of rubber elasticity, to establish a relationship between the chemical changes affecting the macromolecular skeleton (chain scissions or crosslink events) and the elastic properties. In its simplest form, this theory applies to ideal networks in which every chain is connected to the network by its two extremities (Figure 7).

**FIGURE 8:**
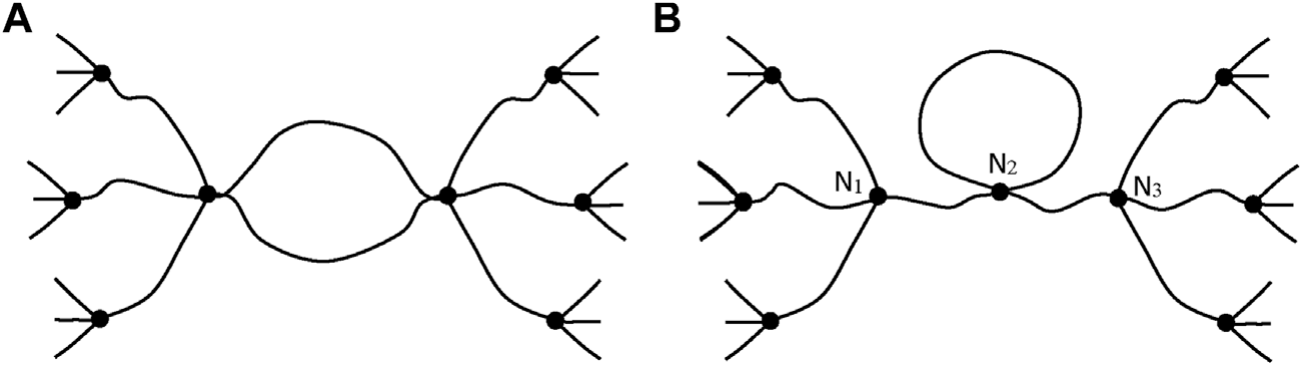
Schematization of cycles for two cases. **(A)** Only one chain, among the two connected to the same crosslinks, is active. **(B)** The junction N_2_ is not a crosslink, there is only one EAC between N_1_ and N_3_ (case of a dangling cycle).

Such chains are named: “elastically active chains” (EAC). If two chains are connected to the same crosslink node (Figure 8), only one can be considered as an EAC.

An ideal network is characterized by the average molar mass $M_{E}$ of EAC, the concentration of EACs being given by:

$$\nu =\frac{1}{M_{E}}$$ (45)

(expressed in mol.kg^−1^)

and the concentration of crosslinks by:

$$n=\frac{2}{f\times \nu }$$ (46)

where *f* is the crosslink functionality, i.e., the number of chains linked to a crosslink.

f=4 in the above figures. The crosslink density can be represented by ν or n . It can also be represented by the product: ν×ρ or n×ρ , i.e., the number of EACs or crosslinks per volume unit, ρ being the rubber density.

Let us now consider the consequences of a chain scission or a crosslinking event (the number of chain scissions or crosslinks created per mass unit being noted *S* or *X*, respectively) on crosslink density.

#### Chain Scissions

##### In a Network Containing Trifunctional Crosslinks

**FIGURE 9:**
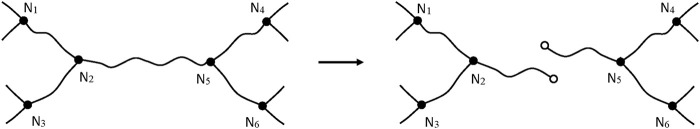
Schematization of a chain scission in a trifunctional network.

After a chain scission, N_2_ and N_5_ lose their crosslink character, the elastically active chains N_1_N_2_ and N_2_N_3_ form a single EAC. In the same way, N_4_N_5_ and N_5_N_6_ form a single EAC. Three EACs are thus lost (Figure 9). It can be thus written:

$$\nu =\nu_{0}-3S$$ (47)

##### In Networks of Functionality Higher Than 3

**FIGURE 10:**
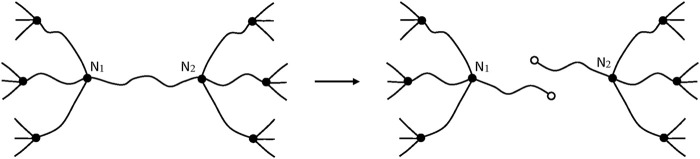
Schematization of a chain scission in a network of functionality higher than 3.

In this case, only one EAC is lost per chain scission (Figure 10), so that:

$$\nu =\nu_{0}-S$$ (48)

The network nodes N_1_ and N_2_, which had initially a functionality *f*, now acquire the new functionality (*f—1*).

##### Selective Scission in Tetrafunctional Networks

**FIGURE 11:**
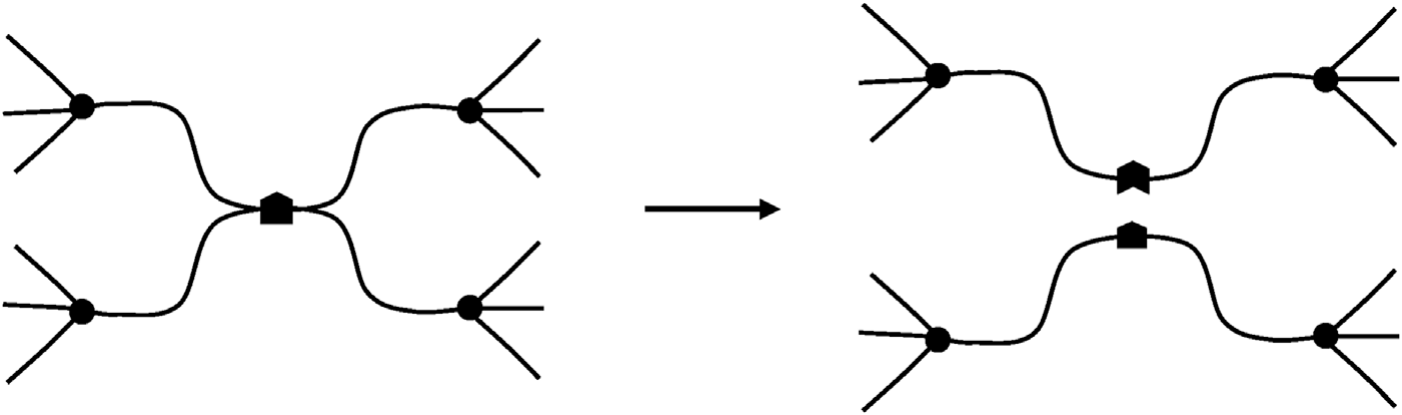
Schematization of a “selective” scission.

Another mode of scission can exist in some tetrafunctional networks (Figure 11). Here, the scission destroys one crosslink without creating dangling chains. It can be written:

$$\nu =\nu_{0}-2\mathrm{SS}$$ (49)

where *SS* is the number of “selective” scissions per mass unit. This process can be called “reversion”.

#### Crosslinking Reactions

**FIGURE 12:**
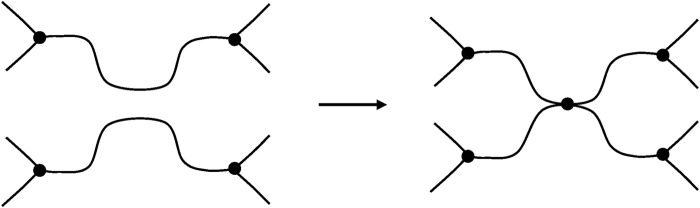
Schematization of a crosslink event in a trifunctional network.

Oxidative crosslinking results always from intermolecular reactions creating a tetrafunctional crosslink. In trifunctional networks (Figure 12), it can be written:

$$\nu =\nu_{0}+2X$$ (50)

Note that this relationship remains true whatever the crosslink functionality.

#### Ideal Network Description

Real networks always contain initially dangling chains (DC) corresponding to the chain ends of the starting linear polymer of number average molar mass $M_{n}$ . The concentration of elastically active chains ν can be estimated at low concentrations of dangling chains by:

$$\nu =\nu_{t}\left(1-\frac{\varphi \times b}{\nu_{t}}\right)$$ (51)

where $\nu_{t}$ is the total chain concentration and *b* is the dangling chain concentration. φ=2 for trifunctional crosslinks, and φ=1 for crosslinks of functionality f>3 .

This relationship can be transformed into:

$$\nu =\frac{1}{M_{E}}\left(1-\frac{2\varphi \times M_{E}}{M_{n}}\right)$$ (52)

where $M_{E}$ is the sub (chain) molar mass such as:

$$\nu_{t}=\frac{1}{M_{E}}$$ (53)

**FIGURE 13:**
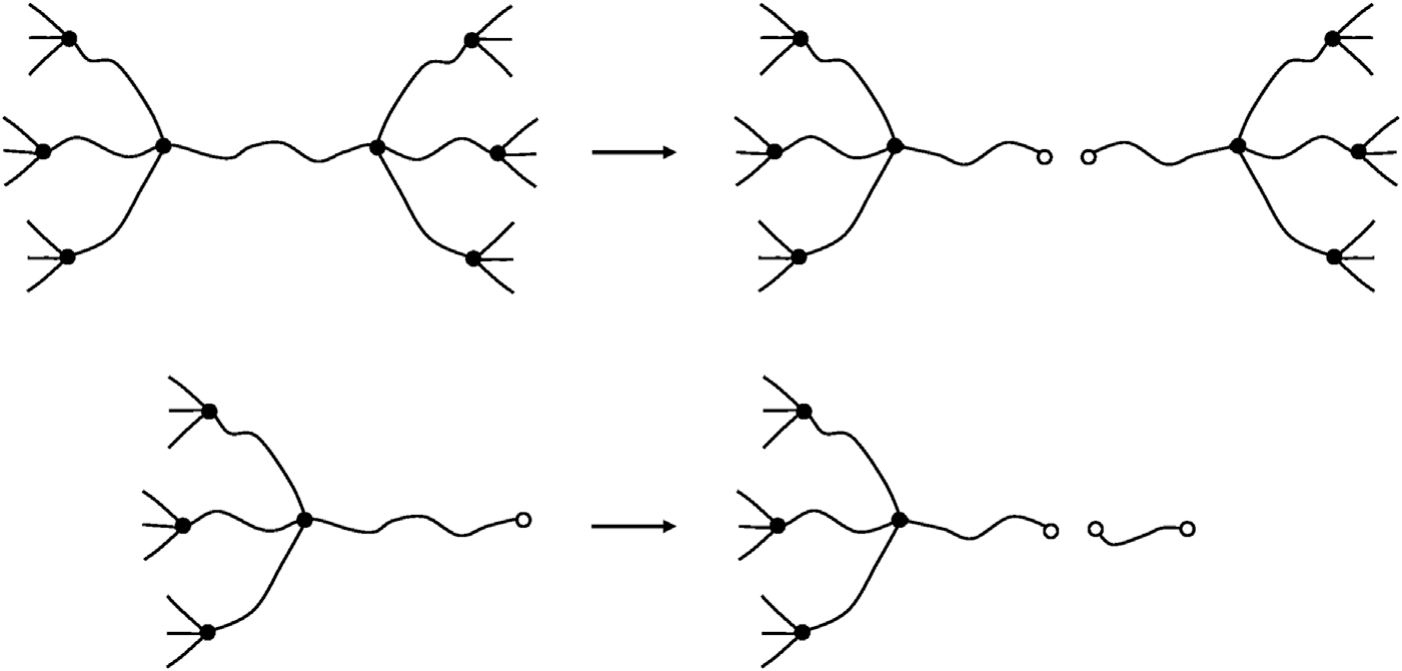
Schematization of a chain scission on an elastically active chain **(top)** and on a dangling chain **(bottom)**.

Let us consider only the case of crosslink functionalities f>3 ( φ=1 ). Chain scissions can occur as well on elastically active chains as in dangling chains (Figure 13).

If the total number of chain scissions per mass unit is *S*, the number of chain scissions on elastically active chains will be:

$$S_{E}=S\frac{\nu }{\nu +b}$$ (54)

and the number of chain scissions on dangling chains will be:

$$S_{D}=S\frac{b}{\nu +b}$$ (55)

These latter create extractible short chains but do not modify, at least in a first approach, the crosslink density. In contrast, scissions on elastically active chains increase the total number of chains:

$$\nu_{t}=\nu_{t0}+S_{E}$$ (56)

and increase the total number of dangling chains:

$$b=b_{0}+2S_{E}$$ (57)

Thus:

$$\nu =\nu_{t}-b=\nu_{0}-S_{E}$$ (58)

Crosslinking can also occur on elastically active chains or on dangling chains. In principle, three possibilities exist. Assuming their equireactivity (with the same rate constant *k*), it can be written:

- EAC + EAC ⇒ $r_{1}=k\times \nu^{2}$

- EAC + DC ⇒ $r_{2}=k\times \nu \times b$

- DC + DC ⇒ $r_{3}=k\times b^{2}$

where $r_{1}$ , $r_{2}$ and $r_{3}$ are the corresponding crosslinking rates.

**FIGURE 14:**
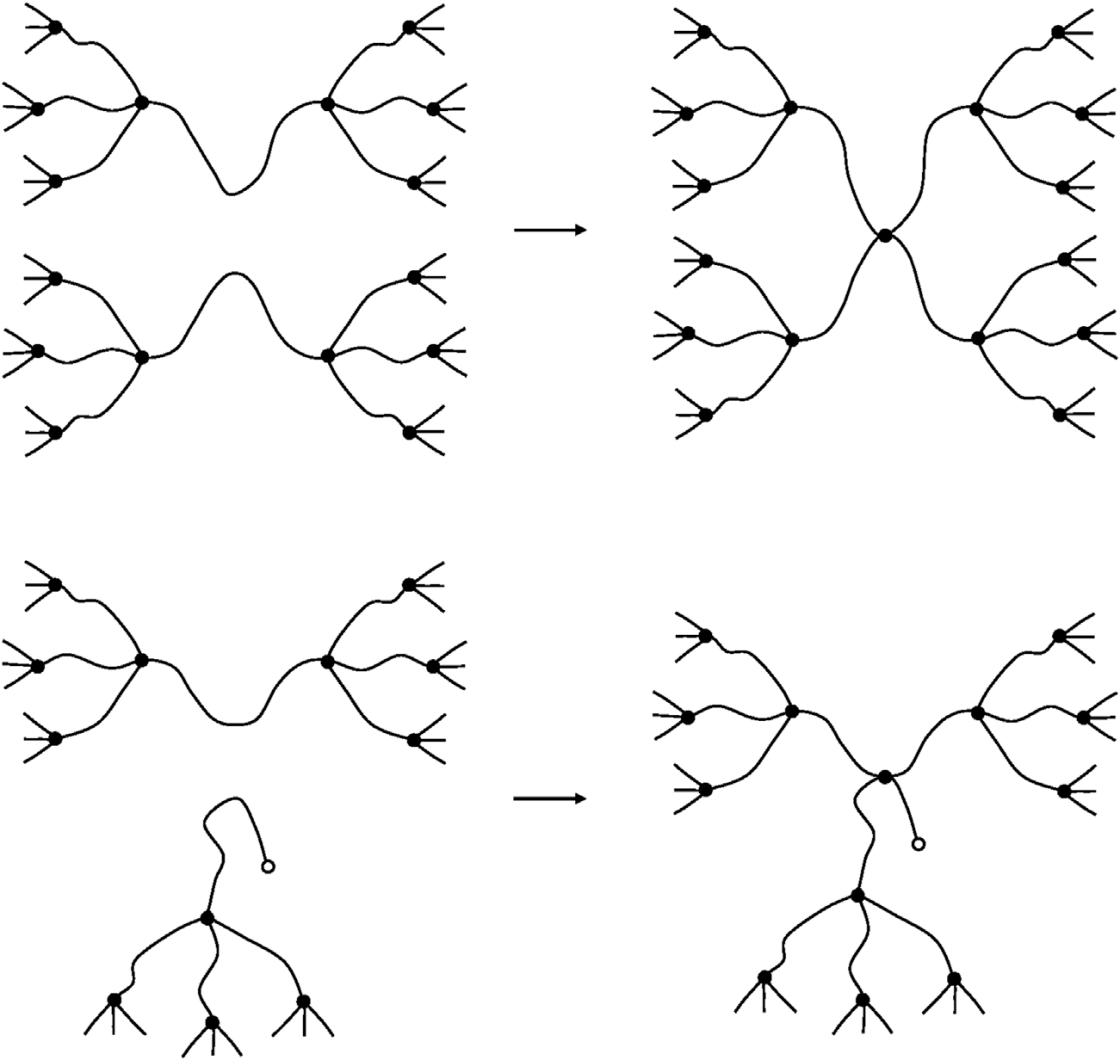
Schematization of a crosslinking reactions EAC + EAC **(top)** and EAC + DC **(bottom)**.

In the case of low concentrations of dangling chains ( b/ν≪1 ), $r_{3}$ can be neglected and only two cases have to be considered (see Figure 14).

It can be observed that, in both cases, two new elastically active chains are created. Thus:

$$\nu =\nu_{0}+2X$$ (59)

Thus, in low concentrations, dangling chains do not affect the crosslinking yield.

#### Consequences of Ageing on Mechanical Properties

The changes in the concentration of EACs can be characterized by mechanical experiments using the theory of rubber elasticity. In the simplest version of this theory, the behaviour law in tension is (Flory, 1953):

$$\sigma =\mathrm{\rho RT\nu}\left(\Lambda^{2}-\Lambda^{-1}\right)$$ (60)

where σ is the stress, Λ the draw ratio and ρ the rubber density.

At small deformations (i.e. Λ−1≪1 ), on obtains:

σ≈3ρRTνε (61)

where ε=Λ−1 is the strain.

The Young’s modulus is thus given by:

E=3ρRTν (62)

and the shear modulus by:

G=ρRTν (63)

knowing that the Poisson’s coefficient is very close to 0.5 (deformation at constant volume).

The concentration of EACs can also be determined from the equilibrium swelling ratio in a solvent (Flory and Rehner, 1943):

$$\nu =\frac{-\mathrm{Ln}\left(1-\vartheta \right)-\vartheta -\chi \vartheta^{2}}{\mathrm{\rho V}\left(\vartheta^{1/3}-\frac{\vartheta }{2}\right)}$$ (64)

where ϑ is the network volume fraction in the network-solvent mixture, V is the solvent molar volume, ρ is the rubber density and χ is the polymer-solvent interaction parameter.

The application of these relationships to oxidized networks can be problematic because rubbers are generally polymers of low polarity. Their oxidation leads to the grafting of oxygenated structures of medium (ketones, esters, etc.) to high (alcohols, carboxylic acids, etc.) polarity. Since the interaction parameter is sharply linked to the polarity and since its variation can affect significantly the value of ϑ , the results should be considered with caution.

Mechanical experiments must then be recommended, but it is not free of difficulties. Indeed, there are more or less sophisticated variants of the rubber elasticity theory, for instance, according to the “phantom theory” (James and Guth, 1943):

G=ρRT(ν−n) (65)

where n is the number of crosslinks (Eq. 46).

But, the most important modification was made by Mooney and Rivlin (Rivlin, 1948; Gumbrell et al., 1953):

$$\sigma =\left(C_{1}+C_{2}\Lambda^{-1}\right)\left(\Lambda^{2}-\Lambda^{-1}\right)$$ (66)

where

$$C_{1}=\mathrm{\rho RT\nu}$$ (67)

The term $C_{2}{\text{Λ}}^{-1}$ was added in Eq. 66 in order to reduce the deviation between the Flory’s law and the experimental data at low deformations. According to some theories (e.g. Rault, 1993), $C_{1}\approx C_{2}$ in dry state, but $C_{1}/C_{2}$ would decrease with the solvent concentration and vanish in highly swollen samples. Although some data are available, the relationship between network structure and $C_{2}$ remains an open issue. At the present state of our knowledge, the basic theory remains the only way to determine ν from elastic properties.

**FIGURE 15:**
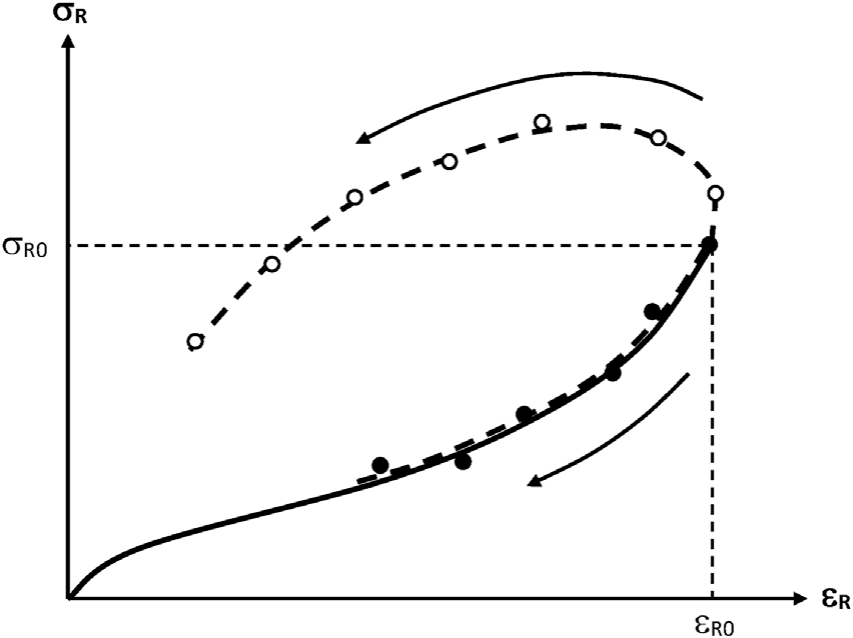
Schematization of the general trend of the rupture envelopes for a predominant chain scission (-●-) or crosslinking process (-O-). The full line represents the initial tensile curve. The arrows indicate the sense of variation of the fracture properties with the time of exposure.

The effect of ageing on the ultimate properties of networks can be summarized by Figure 15, which shows the rupture envelope for rubber samples undergoing a predominant chain scission or crosslinking process. The ultimate properties ( $\sigma_{R}$ , $\varepsilon_{R}$ ) were recorded for each time of exposure, and $\sigma_{R}$ was plotted versus $\varepsilon_{R}$ to obtain the rupture envelope.

##### Predominance of Crosslinking Reactions

When crosslinking predominates, the stress at break increases while the elongation at break decreases with time of exposure. Rubber elasticity theory allows establishing a relationship between the crosslink density *n* and the ultimate elongation $\varepsilon_{R}$ , if making the assumption that the rupture occurs when the elastically active chains reach their maximum elongation.

Let us consider the molar mass $M_{E}$ for the elastically active chains. In the case of tetrafunctional crosslinks, it can be written:

$$n=\frac{1}{2M_{E}}$$ (68)

On the other hand, since the end to end distance of an unstressed chain is proportional to $M_{E}^{1/2}$ , whereas it is proportional to $M_{E}$ for the fully elongated chain, it is easy to show that the draw ratio at break $\Lambda_{R}$ is given by:

$$\Lambda_{R}=1+\varepsilon_{R}=K\times M_{E}^{1/2}$$ (69)

So that:

$$\Lambda_{R}=1+\varepsilon_{R}=K{\left(\frac{1}{2n}\right)}^{1/2}$$ (70)

where *K* is a factor essentially depending on the monomer unit structure.

In principle, $M_{E}$ (i.e. *n*) can be determined from the Young’s modulus values:

$$E=3\frac{\mathrm{\rho RT}}{M_{E}}=\frac{3}{2}\mathrm{\rho RTn}$$ (71)

Thus, in the ideal case:

$$\Lambda_{R}\propto E^{-1/2}$$ (72)

There are many reasons, however, for a deviation from ideality, e.g., heterogeneous distribution of $M_{E}$ , presence of fillers, filler agglomerates, etc.

##### Predominance of Chain Scissions

When chain scissions predominate, both the stress and elongation at break decrease with time of epoxure. In some cases, it was observed that the rupture envelope coincides more or less with the initial tensile curve (Gueguen et al., 1994). What determines embrittlement, here, is the fact that less and less elastically active chains are able to sustain the imposed stress. Then, the stress concentration on the most stressed chains induces their rupture, the stress is redistributed on a smaller number of chains among which some of them become overloaded, etc. Unfortunately, there is no theoretical analysis, to our knowledge, of this process.

Young’s modulus measurements remain the only way to quantitatively analyse oxidative ageing when chain scissions are predominant. As seen above, the number of elastically active chains is given by:

$$\nu =\nu_{0}-S_{E}$$ (73)

where

$$S_{E}=S\frac{\nu }{\nu +b}$$ (74)

*S* being the overall number of chain scissions and *b* the number of dangling chains. Thus:

$$E=3\mathrm{\rho RT}\left(\nu_{0}-S_{E}\right)=E_{0}-3\mathrm{\rho RT}S_{E}$$ (75)

i.e.

$$E=E_{0}-3\mathrm{\rho RTS}\frac{\nu }{\nu +b}$$ (76)

Let us recall that, at low conversions: S≪ν .

### Experimental Results on Radio-Cured Rubbers

The effects of irradiation on rubber structure have previously been described. Some very general features can play an important role on the ageing behaviour.

#### Consumption of Reactive Species

Some reactive species, e.g., double bonds, are preferential sites for radical chain oxidation. Their consumption can thus be responsible for some stability improvement.

#### Consumption of Stabilizers

Chain breaking stabilisers of the amine or phenol type are partially or totally consumed during irradiation. Very simple kinetic models predict that these stabilizers are consumed with a rate equal to the initiation rate, according to the following mechanistic scheme (Emanuel and Buchachenko, 1987):

Initiation:

Polymer (PH) + hν + O_2_ → PO_2_^•^ (*r*_*i*_)

Propagation:

PO_2_^•^ + PH + O_2_ → POOH + PO_2_^•^ (*k*_*3*_)

Stabilization:

PO_2_^•^ + AH → Inactive products (*k*_*S*_)

In the steady state, the termination rate is equal to the initiation rate. It can thus be written:

$$\frac{d\left[\mathrm{AH}\right]}{\mathrm{dt}}=-k_{s}\left[\mathrm{AH}\right]\left[PO_{2}^{•}\right]$$ (77)

and

$$k_{s}\left[\mathrm{AH}\right]\left[PO_{2}^{•}\right]=r_{i}$$ (78)

It comes:

$$\frac{d\left[\mathrm{AH}\right]}{\mathrm{dt}}=-r_{i}$$ (79)

i.e.

$$\left[\mathrm{AH}\right]={\left[\mathrm{AH}\right]}_{0}-r_{i}\times t$$ (80)

The totality of stabiliser is consumed after a time t_S_ such as:

$$t_{S}=\frac{{\left[\mathrm{AH}\right]}_{0}}{r_{i}}$$ (81)

Let us recall that the initiation rate $r_{i}$ is related to the dose rate *I* as follows:

$$r_{i}=10^{-7}G_{i}I$$ (82)

where $G_{i}$ is the radiochemical yield for radical formation. Typical values of $G_{i}$ are of the order 10 molecules per 100 eV in aliphatic polymers (Khelidj et al., 2006a), so that:

$$r_{i}\approx 10^{-6}\times I$$ (83)

The dose for the total disappearance of (chain breaking) antioxidant is thus:

$$D_{S}=I\times t_{S}=10^{6}\times {\left[\mathrm{AH}\right]}_{0}$$ (84)

In rubbers, antioxidant concentrations are of the order of 1 wt%, i.e. for a typical antioxidant having a molar mass of the order of 500 ± 200 g mol^−1^, it is found that: ${\left[\mathrm{AH}\right]}_{0}\approx \left(2\pm 1\right)\times 10^{-1}\mathrm{mol}.kg^{-1}$ . It comes:

$$D_{S}\approx 10^{6}\times \left(2\times 10^{-2}\right)=20\;\mathrm{kGy}$$ (85)

Although the above scheme is questionable in various aspects, it gives realistic predictions. In the presence of oxygen, this scheme allows calculating that almost the totality of antioxidant is consumed by irradiation for the doses commonly encountered in industrial applications.

#### Build-Up of Thermolabile Species (Especially Hydroperoxides)

Hydroperoxides are formed as soon as oxygen is present, even in small quantity. As shown in introduction, hydroperoxide decomposition initiates radical chain oxidation. The induction time is a slowly decreasing function of the initial hydroperoxide concentration. Radio-oxidation is strongly diffusion limited, in particular at high dose rates. In this case, oxidation products are only present in a thin superficial layer of the polymer samples.

#### Crosslinking

Crosslinking reactions are expected to influence transport phenomena: oxygen and stabiliser diffusion rates should be a decreasing function of the crosslinking density. However, in practice, strong variations of the crosslinking density are required to induce significant variations of transport properties (Seguchi and Yamamoto, 1986).

Crosslinking is also expected to modify the fracture mechanisms and thus, to affect the relationship existing between the changes in the molecular structure and the changes in the ultimate mechanical properties.

Data are available on ethylene-propylene-diene (EPDM) terpolymers and on nitrile rubbers (Lopitaux, 2001). These data can be summarized as follows.

#### EPDM Elastomers

Consumption of double bonds of the diene monomer (IR band at 808 cm^−1^) is effectively observed during irradiation, but vinylidene, vinyl and transvinylene double bonds (absorbing at 890, 910 and 967 cm^−1^, respectively) appear as a result, probably, of disproportionation reactions.

**FIGURE 16:**
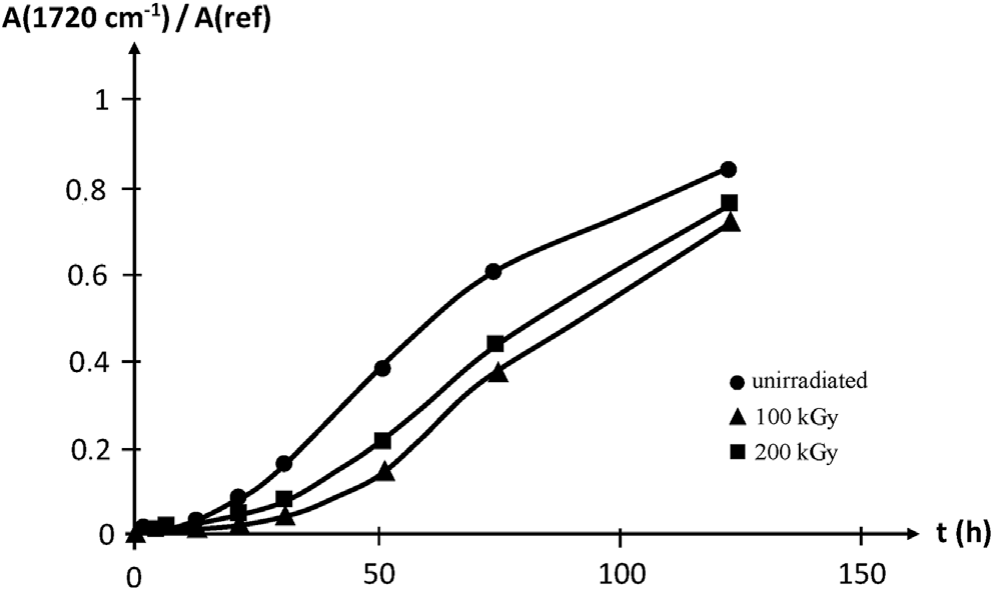
Carbonyl build-up in EPDM samples aged at 100°C in air. Results coming from the PhD dissertation of Lopitaux (2001).

Thermal oxidation at 100°C reveals that irradiation improves slightly, but significantly, the oxidative stability of EPDM, which is attributed to the strong sensitizing effect of the diene monomer unit. Samples irradiated up to 200 kGy are however less stable than those irradiated up to 100 kGy, thus showing that irradiation creates also oxidation sensitive sites, presumably double bonds (Figure 16).

There is no qualitative difference between oxidative ageing behaviours of radiochemically and thermally cured EPDM rubbers:

1) Oxidation displays an induction time of which the duration is a decreasing function of temperature and an increasing function of the concentration and efficiency of stabilisers.
2) During the induction period, a non monotonic variation of the ultimate stress resulting from the competition between chain scissions (due to oxidation) and post-curing (due to the presence of unreacted diene units) can be observed.
3) After the end of the induction period, chain scissions predominate, weight loss and density increase (due to oxygen incorporation into polymer chains) are observed. Chain scissions lead to deep embrittlement, decrease in Young’s modulus and increase in soluble fraction.

#### NBR Elastomers

NBR elastomers are butadiene-acrylonitrile copolymers. In these copolymers, the butadiene part is especially sensitive to oxidation, because of the presence of allylic methylenes, in which hydrogen abstraction is very easy, and double bonds favouring intra as intermolecular addition of radicals. Thus, thermal oxidation of NBR displays similar features as polybutadiene, especially the predominance of a crosslinking over chain scissions (Coquillat et al., 2007b).

**FIGURE 17:**
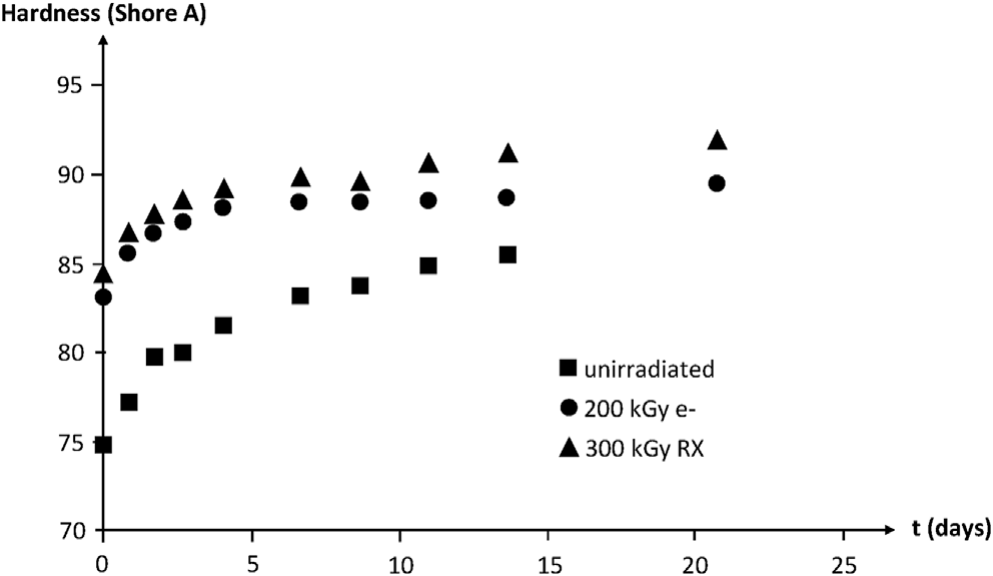
Hardness of NBR samples with various radio-curing doses versus their time of exposure at 130°C in air. Results coming from the PhD dissertation of Lopitaux (2001).

IR spectrophotometry allows putting in evidence the build-up of oxygen containing species (i.e., carbonyls, hydroxyls, etc.). No induction period is observed at 130°C in air owing to the high polymer reactivity towards oxygen. The hardness increase reveals the effect of crosslinking (Figure 17).

**FIGURE 18:**
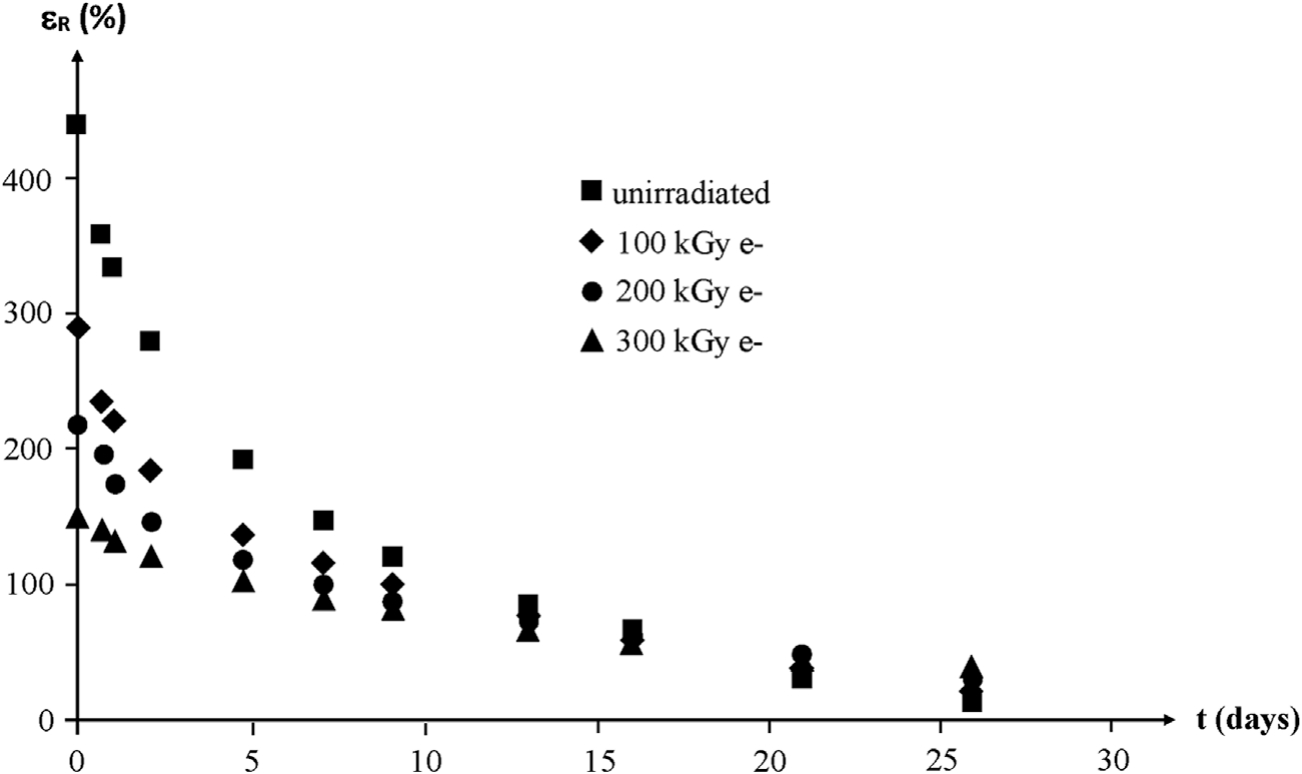
Ultimate elongation of NBR samples with various radio-curing doses versus their time of exposure at 130°C in air. Results coming from the PhD dissertation of Lopitaux (2001).

Whatever their initial structure, the ultimate elongation of NBR samples decreases quasi exponentially to finally reach values lower than 50% after about 1 month of exposure at 130°C in air (Figure 18).

**FIGURE 19:**
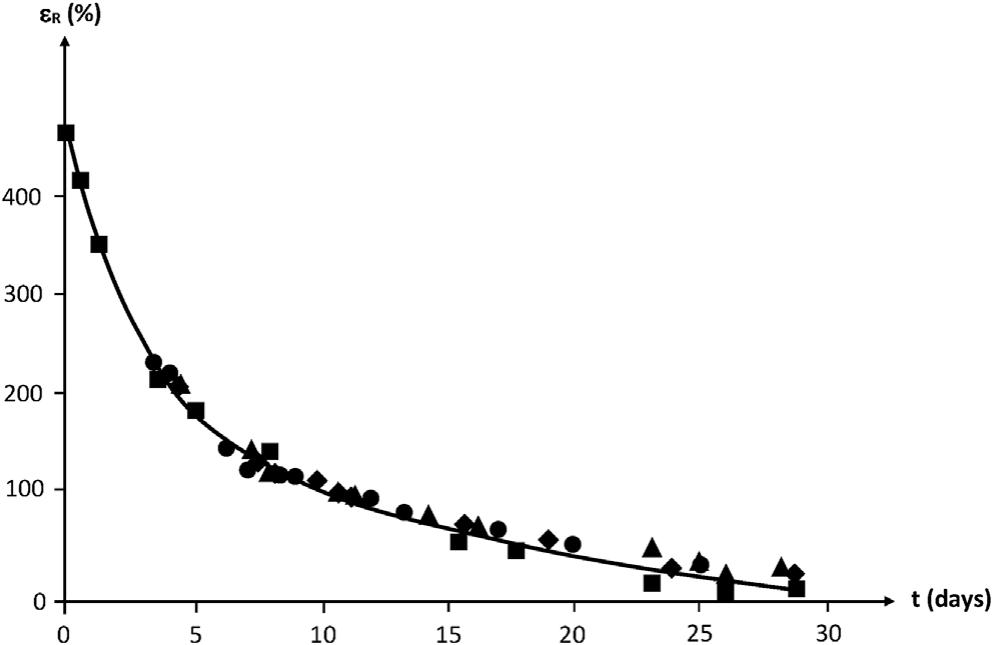
Mastercurve obtained from a horizontal shift of the curves of Figure 18.

By translating the curves along the time axis, a mastercurve can be obtained (Figure 19). Taking the unirradiated sample as a reference, the shift factor is ≈ 2–3 days for 100 kGy, ≈ 4 days for 200 kGy and ≈ 6–7 days for 300 kGy.

**FIGURE 20:**
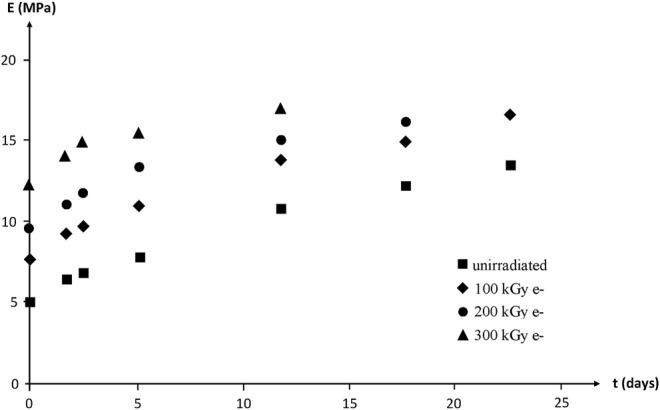
Young’s modulus (secant modulus at Λ=2 ) of NBR samples with various radio-curing doses versus their time of exposure at 130°C in air. Results coming from the PhD dissertation of Lopitaux (2001).

Modulus changes are shown in Figure 20. According to the rubber elasticity theory, it would be expected that:

$$\frac{\Lambda_{R}}{\Lambda_{R0}}={\left(\frac{E_{0}}{E}\right)}^{1/2}$$ (86)

where $\Lambda_{R0}$ , $\Lambda_{R}$ , $E_{0}$ and E are the draw ratio at break and Young’s modulus before and after thermal ageing at 130°C in air, respectively.

In other words, $\Lambda_{R}$ is expected to vary more slowly than E . The deviation from the theory is obvious here:

1) Except for the highest irradiation dose, $\Lambda_{R}$ varies faster than E .
2) Initial variation rates with time of exposure for $\Lambda_{R}$ (or $\varepsilon_{R}$ ) depend sharply on irradiation dose, whereas initial variation rates for E appear independent (within experimental scattering) of irradiation dose. One possible explanation is that E is essentially governed by the number of elastically active chains, i.e., by their average molar mass $M_{E}$ , whereas $\Lambda_{R}$ depends essentially on the fraction of the shortest elastically active chains. As a matter of fact, these latter reach their maximum elongation before the longest ones during the tensile experiment.

These results show the difficulty to build a non-empirical model for lifetime prediction in this domain where some key data, for instance the distribution of the molar masses of the elastically active chains, are practically out of reach at the moment. Consequently, there is no way yet to avoid the introduction of some empirism in the kinetic modelling.

## Conclusion and Prospects

This brief literature overview on two ageing modes of radiation cured polymers: humid ageing of polymer glasses and thermal oxidative ageing of rubbers, led to the following conclusions.

On the one hand, it was shown that radio-cured thermosets of the acrylate type can absorb between 2 and 5 wt% of water at equilibrium in saturated atmosphere. This hydrophilicity is mainly due to the presence of alcoholic groups in the (epoxy-acrylic) structure. The water equilibrium concentration is almost independent of temperature because the heat of dissolution *H*_*S*_ is close to the heat of water vaporization *H*_*W*_, but of opposite sign. It is an increasing function of relative hygrometry (or water activity *a*), and the slope $dW_{e}/da$ is an increasing function of *a*, thus indicating the presence of clusters of small size at high activities. The water diffusivity in matrices is almost independent of relative hygrometry but increases with temperature, its apparent activation energy being of the order of 50 kJ mol^−1^ and the order of magnitude of the diffusion coefficient being of the order of 10^−12^ m^2^ s^−1^ around 70°C. It is 3–5 times lower in carbon fibres reinforced composites with a fibre fraction of 71 ± 5 wt% (Dorbe, 2000).

Water absorption induces plasticization, the decrease in *T*_*g*_ being of the order of 8 K per percent of water absorbed. A slight swelling is expected, but there is, to our knowledge, no experimental data on this aspect.

The first order rate constants of hydrolysis are of the same order of magnitude as those previously determined for aliphatic polyesters, except for methacrylates, which are significantly more stable. Hydrolysis kinetics seems to be unaffected by the presence of carbon fibres in the composites studied by Dorbe (2000). However, it cannot be excluded the eventuality of cases where the composite durability would be controlled by the failure of the fibre-matrix interface rather than the matrix degradation.

Design rules for humid ageing resistant systems can simply be deduced from the expression of the hydrolysis rate:

$$\frac{\mathrm{dS}}{\mathrm{dt}}=k\left[\mathrm{Ester}\right]\left[\mathrm{Water}\right]$$

The rate constant *k* can be decreased replacing acrylic monomers by methacrylic ones. The ester concentration can be decreased by increasing the length of the epoxy chain separating acrylic moieties. The water concentration can be decreased by reducing the number of (or suppressing) hydroxyl groups. However, all these modifications can have negative counterparts, for instance methacrylic esters are less thermostable than acrylic ones; the decrease of acrylic concentration can affect the polymerization rates; the suppression of hydroxyl groups involves radical changes in monomer chemistry. At this stage of the investigations, there is a lack of experimental data to appreciate the real progression margin in this domain.

On the other hand, it was recalled that hydrocarbon rubbers are among the most sensitive polymers to oxidation, especially when they contain double bonds in the monomer unit (i.e., polydienes and their copolymers) or in the crosslinking species (e.g., diene unit in ethylene-propylene-diene (EPDM) terpolymers). Oxidation results from a radical chain mechanism propagating by H abstraction, by radical addition to double bonds or by both processes. In saturated polymers, e.g., in EPDM rubbers, chain scissions tend to predominate. In unsaturated polymers, especially in homo or copolymers of polybutadiene, crosslinking can predominate.

Radio-cured rubbers do not exhibit a thermal oxidative behaviour different from thermally cured rubbers. Consequently, a great part of the very abundant literature published on the thermal ageing of thermally cured rubbers remains exploitable for radio-cured rubbers. Some more or less specific aspects of radiation curing can however be observed:

1) In the early periods of exposure, thermal ageing is often dominated by post-curing reactions. Irradiation consumes more or less reactive crosslinking species and thus, reduces the initial rate and amplitude of thermal post-curing.
2) However, irradiation produces also oxidizable species, especially double bonds resulting from disproportionation reactions. At high dose rates, the stabilizing effect of irradiation due to the consumption of crosslinking species can be counterbalanced by the sensitizing effect of double bonds formed during irradiation. This is probably the reason why the thermal oxidative stability varies non-monotonically with the irradiation dose.
3) Irradiation consumes stabilisers, especially when the material is irradiated in the presence of oxygen. Unfortunately, relatively little is known about these aspects at this moment.

Generally speaking, it appears that radio-curing does not affect catastrophically the thermal stability of hydrocarbon rubbers.

As the ageing of radiochemically and thermally cured polymers are not so different, the research prospects are quite similar for both types of polymers. In addition to the development of more accurate analyses to better understand and define the network structure, the current prospects are: a better understanding of the aging mechanisms and their consequences on the physico-chemical and mechanical properties, the determination of non-empirical structure/property relationships, the determination of structural end-of-life criteria, etc.

Over the past 3 decades, the introduction of numerical methods in the field of polymer ageing has allowed removing many simplifying assumptions. Now, it opens the way for the development of a more efficient and fully scientific approach for the lifetime prediction of polymers. The next few years should probably emerge as the transition period between the empirical approaches, only based on simplistic models (e.g., Arrhenius’ law), and the modern approach rather based on kinetic models that are derived from mechanistic schemes and are interfaced with non-empirical structure/property relationships.
